# STAT2 signaling restricts viral dissemination but drives severe pneumonia in SARS-CoV-2 infected hamsters

**DOI:** 10.1038/s41467-020-19684-y

**Published:** 2020-11-17

**Authors:** Robbert Boudewijns, Hendrik Jan Thibaut, Suzanne J. F. Kaptein, Rong Li, Valentijn Vergote, Laura Seldeslachts, Johan Van Weyenbergh, Carolien De Keyzer, Lindsey Bervoets, Sapna Sharma, Laurens Liesenborghs, Ji Ma, Sander Jansen, Dominique Van Looveren, Thomas Vercruysse, Xinyu Wang, Dirk Jochmans, Erik Martens, Kenny Roose, Dorien De Vlieger, Bert Schepens, Tina Van Buyten, Sofie Jacobs, Yanan Liu, Joan Martí-Carreras, Bert Vanmechelen, Tony Wawina-Bokalanga, Leen Delang, Joana Rocha-Pereira, Lotte Coelmont, Winston Chiu, Pieter Leyssen, Elisabeth Heylen, Dominique Schols, Lanjiao Wang, Lila Close, Jelle Matthijnssens, Marc Van Ranst, Veerle Compernolle, Georg Schramm, Koen Van Laere, Xavier Saelens, Nico Callewaert, Ghislain Opdenakker, Piet Maes, Birgit Weynand, Christopher Cawthorne, Greetje Vande Velde, Zhongde Wang, Johan Neyts, Kai Dallmeier

**Affiliations:** 1 Laboratory of Virology and Chemotherapy, Rega Institute, KU Leuven Department of Microbiology, Immunology and Transplantation, 3000 Leuven, Belgium; 2 Molecular Vaccinology and Vaccine Discovery Group, Leuven, Belgium; 3 GVN, Global Virus Network, Baltimore, MD USA; 4 Translational Platform Virology and Chemotherapy, Leuven, Belgium; 5 Department of Animal, Dairy, and Veterinary Sciences, Utah State University, Logan, UT 84322-4815 USA; 6 Laboratory of Clinical and Epidemiological Virology, Rega Institute, KU Leuven Department of Microbiology, Immunology and Transplantation, 3000 Leuven, Belgium; 7 Zoonotic Infectious Diseases Unit, Leuven, Belgium; 8 KU Leuven Department of Imaging and Pathology, Biomedical MRI and MoSAIC, 3000 Leuven, Belgium; 9 Immunity and Inflammation Research Group, Immunobiology Unit, Rega Institute, KU Leuven Department of Microbiology, Immunology and Transplantation, 3000 Leuven, Belgium; 10 VIB-UGent Center for Medical Biotechnology, VIB, 9052 Ghent, Belgium; 11 Department of Biochemistry and Microbiology, Ghent University, 9052 Ghent, Belgium; 12 Laboratory of Viral Metagenomics, Leuven, Belgium; 13 KU Leuven Department of Laboratory Medicine, University Hospitals Leuven, 3000 Leuven, Belgium; 14 National Reference Center for Respiratory Pathogens and Enteroviruses, 3000 Leuven, Belgium; 15 Leuven University Vaccinology Center (LUVAC), 3000 Leuven, Belgium; 16 Blood Service, Belgian Red Cross Flanders, Mechelen, Belgium; 17 Faculty of Medicine and Health Sciences, Ghent University, Ghent, Belgium; 18 KU Leuven Department of Imaging and Pathology, Nuclear Medicine and Molecular Imaging and MoSAIC, 3000 Leuven, Belgium; 19 Division of Nuclear Medicine, University Hospitals Leuven, 3000 Leuven, Belgium; 20 KU Leuven Department of Imaging and Pathology, Translational Cell and Tissue Research, 3000 Leuven, Belgium; 21 Division of Translational Cell and Tissue Research, Leuven, Belgium

**Keywords:** Infection, Viral infection, Pathogens, SARS-CoV-2

## Abstract

Emergence of SARS-CoV-2 causing COVID-19 has resulted in hundreds of thousands of deaths. In search for key targets of effective therapeutics, robust animal models mimicking COVID-19 in humans are urgently needed. Here, we show that Syrian hamsters, in contrast to mice, are highly permissive to SARS-CoV-2 and develop bronchopneumonia and strong inflammatory responses in the lungs with neutrophil infiltration and edema, further confirmed as consolidations visualized by micro-CT alike in clinical practice. Moreover, we identify an exuberant innate immune response as key player in pathogenesis, in which STAT2 signaling plays a dual role, driving severe lung injury on the one hand, yet restricting systemic virus dissemination on the other. Our results reveal the importance of STAT2-dependent interferon responses in the pathogenesis and virus control during SARS-CoV-2 infection and may help rationalizing new strategies for the treatment of COVID-19 patients.

## Abstract (web summary)

SARS-CoV-2 infection can result in severe lung inflammation and pathology, but host response remains incompletely understood. Here the authors show in Syrian hamsters that STAT2 signaling restricts systemic virus dissemination but also drives severe lung injury, playing a dual role in SARS-CoV-2 infection.

## Introduction

Severe acute respiratory syndrome coronavirus 2 (SARS-CoV-2) belongs to the family of *Coronaviridae*, which contains a large group of viruses that are constantly circulating in animals and humans. Illness in humans caused by coronaviruses is mostly mild and manifested by respiratory or digestive problems as leading symptoms^1^. However, some coronaviruses, such as SARS-CoV-1, Middle East respiratory syndrome-related coronavirus (MERS-CoV) and the recent SARS-CoV-2, have been responsible for serious outbreaks of severe and lethal respiratory disease^2,3^. Unlike the previous outbreaks with SARS-CoV-1 and MERS-CoV, the current SARS-CoV-2 outbreak has evolved as the largest global health threat to humanity in this century.

The unprecedented scale and rapidity of the current pandemic urges the development of efficient vaccines and antiviral and anti-inflammatory drugs. A key requisite in expediting this process are animal models that recapitulate and allow to understand viral pathogenesis. Such models can, hence, be used to identify new drug targets and to rationalize and preclinically assess preventive and therapeutic countermeasures.

Acute respiratory disease caused by SARS-CoV-1 and MERS infections is characterized by a dysregulated inflammatory response in which a delayed type I interferon (IFN) response promotes the accumulation of inflammatory monocyte macrophages^4–6^. The severe lung disease in coronavirus disease 2019 (COVID-19) patients seems to result from a similar overshooting inflammatory response^2,7,8^. However, because even non-human primates do not fully replicate COVID-19^9^, fragmentary information and still no appropriate animal models, though urgently sought, are currently available to address this hypothesis.

In this study, we set out to understand SARS-CoV-2-induced pathogenesis by comparing SARS-CoV-2 infection in mice and hamsters, including in animals with a knockout (KO) in key components of adaptive and innate immunity. We show that mice are barely susceptible to SARS-CoV-2 infection and that viral replication in the lungs of mice is further restricted by type I IFN signaling. We demonstrate that, in contrast to mice, Syrian hamsters are highly permissive to SARS-CoV-2 infection and virus-induced lung pathology. We establish that in hamsters a dysregulated innate immune response is a driving force behind SARS-CoV-2 pathogenesis, in which in particular signal transducer and activator of transcription factor 2 (STAT2)-dependent type I and III IFN signaling plays a dual role: (i) restricting infection and dissemination on one hand but (ii) driving development of severe lung disease on the other.

## Results

### Type I IFN restrict SARS-CoV-2 infection in mice

To address the lack of appropriate animal models, we first compared the effect of SARS-CoV-2 infection in wild-type (WT) mice of different lineages (BALB/c and C57BL/6) and matched transgenic mouse strains with a KO of key components of adaptive and innate immunity. We used an original patient isolate of SARS-CoV-2 (BetaCoV/Belgium/GHB-03021/2020; Supplementary Fig. 1a), passaged on HuH7 and Vero E6 cells and fully characterized by deep sequencing. No adventitious agents could be detected (Supplementary Fig. 1b, c). SARS-CoV-2 virus stocks from cell culture passage 4 (P4) and P6 were used.

**Fig. 1:**
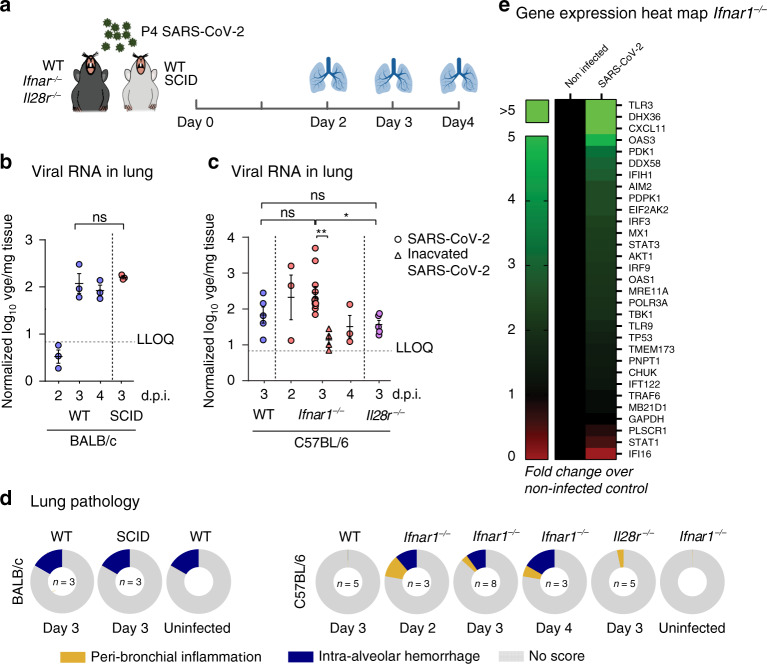
Type I interferon signaling restricts SARS-CoV-2 infection of the lungs of mice. **a** Schematic representation of SARS-CoV-2 inoculation schedule. Several wild-type (WT) and knockout mouse strains were inoculated intranasally with 2 × 10^5^ TCID_50_ of passage 4 (P4) SARS-CoV-2. On the indicated days post inoculation (p.i.), lungs were collected for determination of viral RNA levels and scored for lung damage. **b**, **c** Normalized viral RNA levels in the lungs of BALB/c WT (blue circles, *n* = 3) and SCID (red circles, *n* = 3) mice and C57BL/6 WT (blue circles, *n* = 5), *Ifnar1*^*−/−*^ (day 2 p.i. (red circles, *n* = 3), day 3 p.i. (red circles, *n* = 8), day 3 p.i. inactivated SARS-CoV-2 (red triangles, *n* = 4), day 4 p.i. (red circles, *n* = 3)), and *Il28r*^*−/−*^ (purple circles, *n* = 5) mice. At the indicated time intervals p.i., viral RNA levels were determined by RT-qPCR, normalized against β-actin mRNA levels and transformed to estimate viral genome equivalents (vge) content per weight of the lungs (Supplementary Fig. 2). For heat inactivation, SARS-CoV-2 was incubated for 30 min at 56 °C. Dotted line indicates lower limit of quantification (LLOQ). The data shown are means ± SEM. **d** Histopathological scoring of the lungs for all different mouse strains. Mice were sacrificed on day 3 p.i., and the lungs were stained with H&E and scored for signs of lung damage (inflammation and hemorrhage). Scores are calculated as percentage of the total maximal score. “No score” means not contributing to theoretical full cumulative score of 100%. Numbers (*n*) of animals analyzed per condition are given in the inner circle. **e** Heatmap showing gene expression profiles of 30 selected marker genes in the lungs of uninfected and infected *Ifnar1*^*−/−*^ mice (*n* = 3 per group). Analysis performed on day 3 p.i. The scale represents fold change compared to non-infected animals. Statistical significance between groups was calculated by two-tailed Mann–Whitney *U* test (**b**) or by Kruskal–Wallis with two-sided Dunn’s post hoc test (**c**). *P* values: **P = 0.0013, *P = 0.035 (**c**); ns not significant.

To examine whether adaptive immunity contributed to the susceptibility to SARS-CoV-2 infection, we inoculated WT (immune-competent) and SCID mice (lacking functional T and B cells) from the same BALB/c background intranasally with a high 2 × 10^5^ TCID_50_ (50% tissue culture infectious dose) viral dose (P4 virus) (Fig. 1a). On day 3 post inoculation (p.i.), a viral RNA peak in the lungs was observed (Fig. 1b and Supplementary Fig. 2) with no obvious differences in viral loads (Fig. 1b) or lung pathology (Fig. 1d and Supplementary Fig. 3a, b) between WT and SCID mice. These data indicate that mice that lack the human angiotensin-converting enzyme 2 (ACE2) receptor^10^ can in principle be infected with SARS-CoV-2, although inefficiently and likely transiently, as also observed for SARS-CoV-1^4,11^. However, adaptive immunity did not markedly contribute to this low susceptibility.

IFNs are the prototypic first-line innate immune defense against viral infections. For many respiratory viruses, including SARS-CoV-1, in particular type I and III IFN signaling has been described to play an important role in restricting infection in vivo^12^. To evaluate the role of IFNs in SARS-CoV-2 infection, we compared viral RNA levels and lung pathology in WT C57BL/6 mice and C57BL/6 mice with a genetic ablation of their type I (*Ifnar1*^*−/−*^) and III IFN receptors (*Il28r*^*−/−*^) (Fig. 1a). *Ifnar1*^*−/−*^ mice showed an enhanced replication of SARS-CoV-2 in the lung on day 3 p.i. compared to both WT and *Il28r*^*−/−*^ mice and heat-inactivated inoculum (Fig. 1c). Similar to BALB/c mice, overall viral loads were low with some evidence for an active, although inefficient, virus replication in *Ifnar1*^*−/−*^ mice. In general, mice of all tested strains hardly supported SARS-CoV-2 lung infection, with type I IFN signaling restricting viral replication in vivo.

WT and KO (*Ifnar1*^*−/−*^*, Il28r*^*−/−*^) mouse strains, all on C57BL/6 background, presented consistently with only a mild lung pathology. However, *Ifnar1*^*−/−*^ mice showed increased levels of intra-alveolar hemorrhage, sometimes accompanied by some peri-bronchial inflammation (Fig. 1d and Supplementary Fig. 3a, b). Further evidence for true infection and hence viral replication was provided by transcriptomic analysis^13^ of infected lung tissues (Fig. 1e and Supplementary Fig. 4), revealing (i) a correlation between nucleic acid sensors *TLR3*/*TLR9*/*DDX58*/*POLR3A* and viral load (Supplementary Fig. 4, Cluster 2), (ii) an upregulation of classical antiviral effector molecules^14^ (enrichment *P* < 0.001) such as *cGAS*, *Mx1*, *IFIH1/MDA-5*, *IRF3*, *OAS1*, *OAS3*, and *PKR/EIF2AK2* (Supplementary Fig. 4, Cluster 1), and (iii) downregulation of upstream regulators *STAT1*, *STAT3*, and *STING*/*TMEM173* (Supplementary Fig. 4, Cluster 3), in agreement with a possible role for apoptosis^15–17^. In summary, our data are in line with restriction of SARS-CoV-2 infection by the IFN system in mice, as well as suggest limited inflammatory responses in the lungs of mice, in contrast to COVID-19 in humans^18^. Taken together, mice were considered as a poor model to study COVID-19 pathogenesis or to assess the efficacy of vaccines and treatments.

### STAT2 signaling restricts SARS-CoV-2 replication in hamsters

**Fig. 2:**
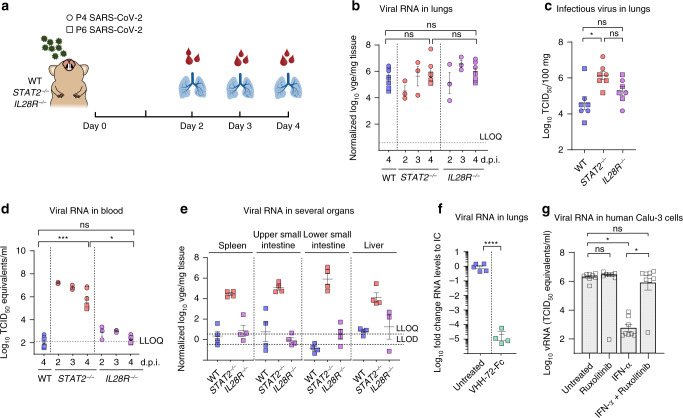
Type I and III interferon signaling restricts SARS-CoV-2 replication in hamsters. **a** Schematic representation of SARS-CoV-2 inoculation schedule. WT (blue), *STAT2*^*−/−*^ (red), and *IL28R-a*^*−/−*^ (purple) hamster strains were inoculated intranasally with 2 × 10^5^ TCID_50_ of passage 4 or 2 × 10^6^ of passage 6 SARS-CoV-2. Outcomes derived from inoculation with passage 4 or passage 6 SARS-CoV-2 is designated by circles (P4, *n* = 3) or squares (P6, *n* = 4). On the indicated days post inoculation (p.i.), organs and blood were collected to determine viral RNA levels and infectious viral load. Viral loads in the indicated organs were quantified by RT-qPCR (**b**, **d**–**f**) or virus titration (**c**). **b**, **e** Viral RNA levels in the lungs (day 2 and day 3 p.i. of each genotype (*n* = 3); day 4 p.i. of each genotype (*n* = 7)) (**b**) or the indicated organs on day 4 p.i. (*n* = 4 for each genotype) (**e**) were normalized against β-actin mRNA levels and transformed to estimate viral genome equivalents (vge) content per weight of the tissue (Fig. S5). **c** Infectious viral loads in the lung on day 4 p.i. are expressed as the number of infectious virus particles per 100 mg of lung tissue (*n* = 7 for each genotype). **d** Viral RNA levels in the blood (day 2 and day 3 p.i. of each genotype (*n* = 3); day 4 p.i. of each genotype (*n* = 7)) were calculated from a standard of infectious virus and expressed as TCID_50_ equivalents per ml blood. Dotted lines indicate lower limit of quantification (LLOQ) or lower limit of detection (LLOD). **f** Viral RNA levels in hamsters after treatment with a previously described single-domain antibody. Hamsters were either left untreated (blue, *n* = 5) or treated with VHH-72-Fc (green, *n* = 4) and sacrificed on day 4 p.i. Viral RNA levels were determined in the lungs, normalized against β-actin, and fold changes were calculated using the 2^(−ΔΔCq)^ method compared to the mean of untreated control. **g** Inhibition of JAK/STAT signaling by Ruxolitinib can rescue SARS-CoV-2 virus replication in human airway epithelial cells from the antiviral effect of type I IFN. Calu-3 (human airway epithelial) cells were left untreated or treated with Ruxolitinib (4 µM), IFN-α (10 IU/ml), or a combination of both (*n* = 8 for each condition). Treatment was initiated 4 h before infection and was continued through the whole experiment. Cultures were infected with P6 SARS-CoV-2 (MOI of 0.1), and 48 h p.i., cell culture supernatant was collected, RNA was extracted, and the amount of vRNA was quantified using RT-qPCR. A serial dilution of the same virus stock was used to generate a standard curve for absolute quantification. The data shown are mean ± SEM. Statistical significance between groups was calculated by Kruskal–Wallis with two-sided Dunn’s post hoc test (**b**–**d**, **g**) or by an unpaired two-sided *t* test (**f**). *P* values: *P = 0.010 (**c**), ***P = 0.0009 and *P = 0.02 (**d**), ****P < 0.0001 (**f**), *P = 0.022 and *P = 0.013 (left to right in **g**); ns not significant.

In contrast to mice, Syrian hamsters have been reported to be highly susceptible to SARS-CoV-1^19^ and SARS-CoV-2^20–22^ infection and might thus provide a useful small animal model to study the involvement of immune responses in restricting SARS-CoV-2 infection and on SARS‑CoV-2-induced lung disease. Intranasal inoculation of SARS-CoV-2 in WT hamsters resulted indeed consistently in high viral RNA loads (Fig. 2b), a proxy used for the quantification of viral loads in the lungs (Supplementary Fig. 5), i.e., roughly 3–4 Log_10_ higher than in *Ifnar1*^*−/−*^ mice (Fig. 1c), as well as high infectious titers (Fig. 2c). Pretreatment with a neutralizing SARS-CoV-1 and SARS-CoV-2-specific single-domain antibody Fc fusion construct (VHH-72-Fc)^23^ reduced viral loads ~10^5^-fold in the lung of WT hamsters, further confirming an active and efficient virus replication and validating hamsters for testing of therapeutic interventions (Fig. 2f). Little, if any, viral RNA was detectable in the blood (Fig. 2d), spleen, upper small intestine, and liver of infected WT hamsters (Fig. 2e).

In order to investigate the roles of type I and III IFN during SARS-CoV-2 infection and pathogenesis, we compared virus replication levels in different tissues of WT hamsters and hamsters with ablated *STAT2* (*STAT2*^*−/−*^ lacking type I and III IFN signaling)^24,25^ and IL28R expression (*IL28R-a*^*−/−*^ lacking type III IFN signaling) (Fig. 2a). Alike in WT hamsters, no signs of overt disease were recognized. Also, no marked differences were observed in viral RNA levels in the lung of WT, *STAT2*^*−/−*^, or *IL28R-a*^*−/−*^ hamsters (Fig. 2b). However, in contrast to WT hamsters, *STAT2*^*−/−*^ hamsters had significantly higher titers of infectious virus in the lung (~50-fold; Fig. 2c), along with very high levels of viral RNA detectable in blood (Fig. 2d), spleen, liver, and their upper and lower gastrointestinal tract^26^ (Fig. 2e). Together, these data suggest that STAT2 is critical for keeping virus replication and production of infectious progeny in the lungs down, as well as for restricting systemic SARS-CoV-2 spread and suppressing viral replication outside of the lung compartment. In line with a strong antiviral activity of STAT2 in hamsters in vivo, a similar effect was also observed in human Calu-3 cells in vitro where pharmacological inhibition of Janus-activated kinase (Jak)/STAT signaling by Ruxolitinib^27,28^ could fully rescue virus replication from the strong inhibitory activity of type I IFN (Fig. 2g and Supplementary Fig. 6), further underscoring the role of STAT2 in controlling virus replication, including in the human model.

### STAT2 signaling drives SARS-CoV-2-induced lung disease

**Fig. 3:**
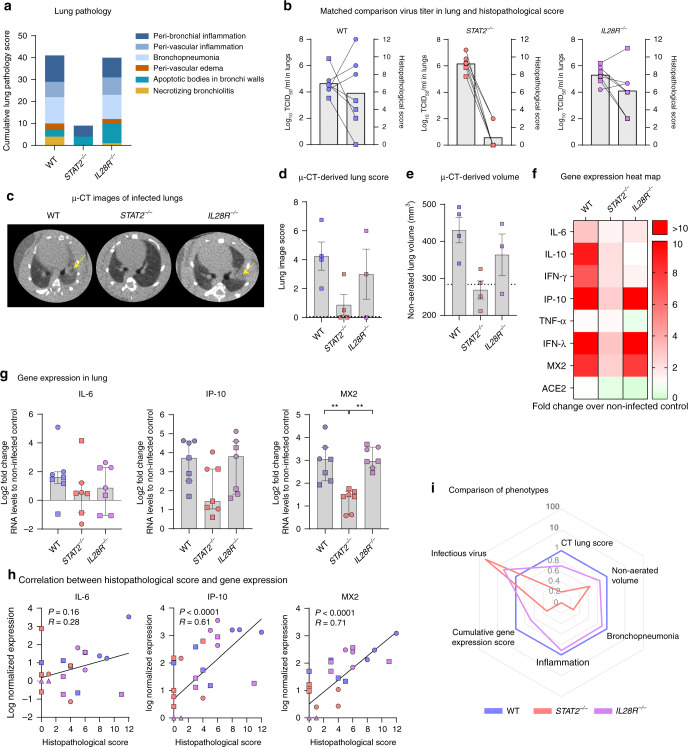
Exuberant innate response by *STAT2* drives SARS-CoV-2-induced lung pathology in hamsters. WT (blue), *STAT2*^*−/−*^ (red), and *IL28R-a*^*−/−*^ (purple) hamster strains were inoculated intranasally with 2 × 10^5^ TCID_50_ of passage 4 (circles, *n* = 3) or 2 × 10^6^ of passage 6 (squares, *n* = 4) SARS-CoV-2. On day 4 p.i., lungs and blood were collected to score for lung damage and determine gene expression levels. **a** Cumulative lung pathology scores for signs of damage. Lungs were stained with H&E and scored for signs of inflammation, bronchopneumonia, edema, apoptotic bodies, and necrotizing bronchiolitis (*n* = 7 for each genotype). **b** Matched comparison between infectious viral load in the lung (left *Y*-axis) (values from Fig. 2c) and histopathological scores (right *Y*-axis) (values used in **a**). Lines indicate matched samples (*n* = 7 for each genotype). **c**–**e** Micro-CT to score for signs of damage in infected (P6 SARS-CoV-2) WT (*n* = 4), *STAT2*^*−/−*^ (*n* = 4), and *IL28R-a*^*−/−*^ (*n* = 3) hamster lungs. **c** Representative transversal micro-CT images. Yellow arrows indicate examples of pulmonary infiltrates seen as consolidation of lung parenchyma. **d** Five transverse cross-sections at different positions in the lung were selected for each animal and scored to quantify lung consolidations. **e** Quantification of the micro-CT-derived non-aerated lung volume biomarker, reflecting the volume of consolidations in the lungs. The data shown are mean ± SEM and lines indicate healthy animals (*n* = 3) (**d**, **e**). **f**, **g** Heat map or individual expression profiles showing differential expression of selected antiviral, pro-inflammatory, and cytokine genes in the lungs after SARS-CoV-2 infection (*n* = 7 per group) relative to non-infected genotype matched controls (WT (*n* = 7), *STAT2*^*−/−*^ (*n* = 3), and *IL28R-a*^*−/−*^ (*n* = 3)). RNA levels were determined by RT-qPCR on lung extracts, normalized for β-actin mRNA levels, and fold changes over the median of uninfected controls were calculated using the 2^(−ΔΔCq)^ method. Only for IFN-λ, where all uninfected control animals had undetectable RNA levels, fold changes were calculated over the lowest detectable value. Data presented as fold change (**f**) or log_2_ fold change over non-infected control (**g**) Bars represent median ± IQR. **h** Correlation between histopathological scores (derived from Fig. 3a) and natural log-normalized gene expression levels (derived from Fig. 3f, g and Supplementary Fig. 9) in uninfected and infected animals. Non-infected animals are indicated by triangles. **i** A spider-web plot showing lung pathology scores (CT lung score, non-aerated lung volume, bronchopneumonia, and inflammation), cumulative gene expression level scores, and infectious virus levels normalized to SARS-CoV-2-infected WT hamsters. Statistical significance between genotypes was calculated by Kruskal–Wallis with two-sided Dunn’s post hoc test (**d**, **e**, **g**) and Spearman correlation (**h**). *P* values: **P = 0.0082 and **P = 0.0025 (left to right in **g**).

Next, we used these different KO hamsters to evaluate the effect of SARS-CoV-2 immune responses on lung pathology. In infected WT hamsters, a marked lung pathology characterized by an infiltration of polymorphonuclear leukocytes, bronchopneumonia, and edema was observed (Fig. 3a and Supplementary Fig. 7). This resembles histopathological findings in humans suffering from severe COVID-19 bronchopneumonia^29^. Inversely, the observed lung pathology was much attenuated in *STAT2*^*−/−*^ hamsters (Fig. 3a). On the contrary, *IL28R-a*^*−/−*^ hamsters showed clear signs of bronchopneumonia and peri-bronchiolar inflammation, similar to those observed in WT animals (Fig. 3a). In general, matching severity of lung pathology with the respective viral loads in the lungs (Fig. 3b) reveals an obvious link between infection and disease for WT and *IL28R-a*^*−/−*^ hamsters, despite variability in individual severity scores. Much by contrast, this link is disrupted conjointly with the ablation of type I IFN responses downstream of STAT2 signaling, in face of unrestricted virus replication.

A similar picture is unveiled when assessing lung pathology by computed tomography (CT). CT imaging was established here by the lack of readily accessible serum markers and absence of overt disease symptoms as a non-invasive means to score for SARS-CoV-2-induced lung disease, alike in standard patient care to aid COVID-19 diagnosis with high sensitivity and monitor progression/recovery^7,8,30,31^. Similar as in humans^32^, semi-quantitative lung pathology scores were obtained from high-resolution chest micro-CT scans of free-breathing animals^33^. Pulmonary consolidations were present in SARS-CoV-2-infected WT and *IL28R-a*^*−/−*^ hamsters but not in *STAT2*^*−/−*^ hamsters (Fig. 3c and Supplementary Fig. 8). Apart from lung consolidations and airway dilation as main observed pathology, marked differences in other micro-CT-derived markers of specific lung pathology, such as hyperinflation, emphysema, or atelectasis^34,35^, were not observed, except in one animal that presented with hyperinflation (Supplementary Fig. 8c). Again, *STAT2*^*−/−*^ hamsters showed minimal lung involvement (Fig. 3c). Further quantitative analysis^34^ revealed an increase of the non-aerated lung volume in SARS-CoV-2-infected WT and *IL28R-a*^*−/−*^ hamsters, yet again not in *STAT2*^*−/−*^ hamsters (Fig. 3d and Supplementary Fig. 8e). First, these data fully support micro-CT as a convenient adjunct to histological scoring (Fig. 3d, e and Supplementary Fig. 8a–e) to visualize and quantify SARS-CoV-2-induced lung injury in the hamster model. Moreover, it may allow to monitor the impact of therapeutic measures non-invasively during disease progression. Second, of relevance for COVID-19 immunopathogenesis, virus replication was unlinked from lung disease in *STAT2*^*−/−*^ hamsters (Supplementary Fig. 8f).

An elevation of serum levels of several cytokines such as interleukin (IL)-6, IL-10, and IFN-γ is reported in critically ill COVID-19 patients^2,7,8^. Intriguingly, these genes were also induced in the lungs of infected hamsters (Fig. 3f, g and Supplementary Fig. 9a), along with elevated mRNA levels of IP-10 (CXCL-10) as reported for other cytokines/chemokines downstream of IFN-γ^20^, and of MX-2 downstream of type I and III IFNs. However, WT, *STAT2*^*−/−*^, and *IL28R-a*^*−/−*^ hamsters each showed marked differences in the respective expression levels (Fig. 3f), with overall strongest induction in WT hamsters. First of all, IFN-stimulated genes^36^ such as MX-2 (strongly induced by type I IFN/STAT2 signaling) and IP-10 (induced by both type I and type II IFNs) showed a differential expression pattern when comparing the different genotypes, triggered by SARS-CoV-2 infection. Lower baseline expression of MX-2 and IP-10 and failure to respond to SARS-CoV-2 infection by MX-2 upregulation in *STAT2*^*−/−*^ hamsters confirmed the functional KO (Supplementary Fig. 9a). As expected, *IL28R-a*^*−/−*^ hamsters showed an intermediate phenotype between that of WT and *STAT2*^*−/−*^ concerning their antiviral IFN response. Of note, the receptor KOs did not affect ACE2 expression^37,38^ in the hamster lungs (Supplementary Fig. 9a). Importantly, the expression levels of IP-10 and MX-2, and to a lesser extent also of IL-6 (Fig. 3g, h; for other possible molecular markers, see Supplementary Fig. 9b), correlated with the disease severities observed in the respective hamster strains (Fig. 3a, c–e), with lowest inflammatory and antiviral cytokine responses in the absence of STAT2-dependent type I and III IFN signaling.

## Discussion

For the development of efficient therapeutic interventions against SARS-CoV-2, relevant small animal models are needed that mimic the different clinical manifestations of COVID-19 and that provide fundamental mechanistic insight into the underlying pathology/pathogenesis. Transgenic mice expressing *hACE2*, the bona fide receptor of SARS-CoV-1 and SARS-CoV-2^39^, have been suggested as COVID-19 model^40–42^. We here demonstrate that WT mice are also susceptible to SARS-CoV-2 infection, yet resulting in very limited viral replication and inflammatory responses. Ablation of type I IFN signaling in *Ifnar1*^*−/−*^ mice results in the same small incremental 10-fold increase in viral replication as was originally reported for SARS-CoV-1^39,43^ in *hACE2* transgenic mice, though recent data suggest an approximate 100-fold increase for SARS-CoV-2 in such models^40,42^. Nevertheless, neither mouse model fully recapitulates pathogenesis of COVID-19 nor allows the study of clinical SARS-CoV-2 isolates without prior adaptation^44^. It remains of interest to investigate the added effect of both (i) *hACE2* knock-in, and (ii) *Ifnar* and/or *Il28r* KO in mice^12,45^. In fact, in WT mice that had been transduced using adenoviral vectors expressing hACE2 to facilitate SARS-CoV-2 infection, concomitant blockage of IFNAR by receptor-neutralizing antibodies resulted in enhanced disease^46^.

In contrast to mice, SARS-CoV-2 infection and associated pathology in WT hamsters seems to resemble what has been reported for SARS-CoV-1 in the same model, confirmed in the meantime also by others^20–22^. An early peak of active virus replication was noted in the lungs with viremia and extra-pulmonary spread. This was accompanied by a strong acute inflammatory response^19^ (as visualized by histopathology) with induction of IL-6, IL-10, and IFN-γ expression (albeit with some variation between animals) like in humans^2,7,8,47,48^ and, as a consequence, pulmonary damage that could readily be depicted as tissue consolidations and obstruction of the lungs by chest micro-CT as established in this study. Micro-CT may hence become a key instrument to non-invasively and quantitatively monitor SARS-CoV-2 lung disease. This will allow to conveniently monitor the effect of therapeutic strategies and test the preclinical efficacy of vaccine candidates.

In humans, SARS-CoV-2 infection begins frequently with a flu-like illness, followed in a second phase by an intense inflammatory response characterized by a cytokine storm and acute lung injury (ALI) and respiratory distress syndrome (ARDS) associated with high mortality^2,7,8^. Considering the very acute and short-lived infection kinetics observed in intranasally inoculated hamsters^20,21^ that may not allow for full development of protracted (bi-phasic) COVID-19-like disease^22^, we studied SARS-CoV-2-induced pathology as early as 4 days p.i. Nevertheless, main pathological findings seem to resemble to a large extent what is observed in humans. Some variability in infection outcome, in particular in pathology scores of WT hamsters (Fig. 3b, h and Supplementary Fig. 8f), may be explained by two experimental constraints. First, the genetics of WT hamsters that are outbred and may hence show a varying susceptibility, as hypothesized in humans^27^. Second, the use of SARS-CoV-2 stocks from two passages P4 and P6 virus stocks. Sequencing analysis of the virus stocks revealed two small in-frame deletions in Spike (S) glycoprotein (i.e., in the N-terminal domain and the furin-cleavage site^49–51^; Supplementary Fig. 1b, c) to dominate in the P6 virus used, likely as a consequence of adaptation to enhanced growth in Vero E6 cells in vitro. This observation is in line with earlier reports, namely, of tissue culture-adaptive mutations similar to those observed by us (Supplementary Fig. 1b, c) resulting in reduced fitness of SARS-CoV-2 in WT hamsters (no body weight loss and attenuation of pathology)^52^, emphasizing possible consequences of using cell culture-adapted virus when studying SARS-CoV-2 infection in animal models.

By using unique KO hamster lines, we demonstrated that STAT2 plays a critical role in mediating antiviral responses and restricting systemic dissemination of SARS-CoV-2 (see Fig. 3i for a summary of phenotypes). This is in line with the effect of STAT1 in a mouse model of SARS-CoV-1 infection^53^. However and much in contrast to what is generally observed for viral infections in *Stat2*^*−/−*^ mice^54^ or *STAT2*^*−/−*^ hamsters^25,55,56^, the severe pathology induced by SARS-CoV-2 in WT hamsters is not observed in the absence of STAT2. Indeed, pneumonia as assessed by sensitive micro-CT was absent in *STAT2*^*−/−*^ hamsters. Considering the established negative regulation of IL-6 and other mediators of inflammation by STAT2^54,57^, our hamster model may help to understand the immune pathogenesis of ALI and ARDS caused by highly pathogenic coronaviruses^12,19,58^ as well as other respiratory viruses^4^. Of note, a meta-analysis of infected human cells revealed a specific SARS-CoV-2-regulated transcriptional footprint for STAT2 in particular that was stronger than that of any other inflammatory transcription factor^59^.

The increase in replication of SARS-CoV-2 seen in *IL28R-a*^*−/−*^ hamsters, on one hand, combined with a tempered inflammatory response and lung injury as compared to WT hamsters, on the other hand, is in line with the role type III IFN plays during respiratory virus infections, including in SARS-CoV-1^45^. This observation also suggests that in humans pegylated IFN-lambda^60,61^ (or similar modulators of innate immunity) may possibly be considered to protect medical staff and other frontline workers from SARS-CoV-2 infection or to dampen symptoms in critically ill patients^62^, however, with caution considering a potential pathophysiological role of IFN-lambda^63,64^.

In conclusion, hamsters may be preferred above mice as infection model for the preclinical assessment of antiviral therapies, of vaccines, and of approaches that aim at tempering the COVID-19 immune pathogenesis in critically ill patients^18,65^. The latter may be achieved by repurposing anti-inflammatory drugs^66^ such as IL-6 receptor antagonists (e.g., Tocilizumab)^67–70^ or small molecule Jak/STAT inhibitors (e.g., Ruxolitinib^71,72^ or Tofacitinib). Educated by our finding that STAT2 signaling plays a dual role in also limiting viral dissemination, targeting the virus-induced cytokine response and overshooting of macrophage activation may need to be complemented by (directly acting) antivirals^73^.

## Methods

### Animals

WT Syrian hamsters (*Mesocricetus auratus*) were purchased from Janvier Laboratories. All other mouse (C57BL/6, *Ifnar1*^*−/−*^, *Il28r*^*−/−*^, BALB/c, and SCID) and hamster (*STAT2*^*−/−*^ and *IL28R-a*^*−/−*^) strains were bred in-house. Six-to-8-week-old female mice and female WT hamsters were used throughout the study. KO hamsters were used upon availability: 7–12-week-old female *STAT2*^*−/−*^ hamsters and 5–7-week-old *IL28R-a*^*−/−*^ hamsters.

*Ifnar1*^*−/−*^ mouse breeding couples were a generous gift of Dr. Claude Libert, IRC/VIB, University of Ghent, Belgium. *Il28r*^*−/−*^ mice [C57B/6N-A<tm1Brd>Ifnlr1<tm1a(EUCOMM)Wtsi>/Wtsi, strain ID: EM:07988] were provided by the Wellcome Trust Sanger Institute Mouse Genetics Project (Sanger MGP)^74^.

*STAT2*^*−/−*^ and *IL28R-a*^*−/−*^ hamsters were generated by CRISPR/Cas-mediated gene targeting. To ablate *STAT2* (Gene ID: 101830537) expression, a 1-nt frameshift mutation was introduced in exon 4 resulting in multiple premature stop codons^24^; to ablate *IL28R* (*IFNLR1;* Gene ID: 101833778) expression, a 22-nucleotide deletion was introduced in exon 2 resulting in multiple premature stop codons in the original open reading frame.

Animals were housed individually (hamsters) or per 5 (mice) in individually ventilated isolator cages (IsoCage N Biocontainment System, Tecniplast) at a temperature of 21 °C, humidity of 55%, and 12:12 dark/light cycles, with access to food and water ad libitum, and cage enrichment (cotton and cardboard play tunnels for mice, wood block for hamsters). Housing conditions and experimental procedures were approved by the ethical committee of KU Leuven (license P015-2020), following institutional guidelines approved by the Federation of European Laboratory Animal Science Associations (FELASA). Animals were euthanized by 100 µl (mice) or 500 µl (hamsters) of intraperitoneally (i.p.) administered Dolethal (200 mg/ml sodium pentobarbital, Vétoquinol SA). Animals were monitored daily for signs of disease (lethargy, heavy breathing, or ruffled fur).

Prior to infection, the animals were anesthetized by i.p. injection of a xylazine (16 mg/kg, XYL-M®, V.M.D.), ketamine (40 mg/kg, Nimatek, EuroVet), and atropine (0.2 mg/kg, Sterop) solution. Each animal was inoculated intranasally by gently adding 50 µl droplets of virus stock containing 2 × 10^5^ TCID_50_ (P4 virus) or 2 × 10^6^ TCID_50_ (P6 virus) in both nostrils. Uninfected animals did not receive any virus or matrix. Due to time constraints by the pandemic outbreak and the urgency to develop a small animal model, we have used virus stock from two different passages (P4 and P6) to characterize our model. A full genotypic characterization of the viruses used is provided in Supplementary Fig. 1, and phenotypic differences and virus titers are provided in the text and legends.

### Cells, virus, and sera

Vero E6 (African green monkey kidney, kind gift from Peter Bredenbeek, LUMC, NL) and HuH7 (human hepatoma, JCRB0403) cells were maintained in minimal essential medium (Gibco) supplemented with 10% fetal bovine serum (Integro), 1% bicarbonate (Gibco), and 1% L-glutamine (Gibco). For maintenance of Calu-3 cells (human airway epithelium, kind gift from Lieve Naesens, KU Leuven, BE), the above medium was supplemented with 10 mM HEPES (Gibco). All assays involving virus growth were performed using 2% (Vero E6 and HuH7) or 0.2% (Calu-3) fetal bovine serum instead of 10%.

SARS-CoV-2 strain BetaCov/Belgium/GHB-03021/2020 (EPI ISL 407976|2020-02-03) recovered from a nasopharyngeal swab taken from a reverse transcription quantitative polymerase chain reaction (RT-qPCR)-confirmed asymptomatic patient returning from Wuhan, China beginning of February 2020^75^ was directly sequenced on a MinION platform (Oxford Nanopore)^76^. Phylogenetic analysis confirmed a close relation with the prototypic Wuhan-Hu-1 2019-nCoV (GenBank accession number MN908947.3) strain. Infectious virus was isolated by serial passaging on HuH7 and Vero E6 cells (see Supplementary Fig. 1), with the addition of penicillin/streptomycin, gentamicin, and amphotericin B. Virus used for animal experiments was from passages P4 and P6. Prior to inoculation of animals, virus stocks were confirmed to be free of mycoplasma (PlasmoTest, InvivoGen) and other adventitious agents by deep sequencing on a MiSeq platform (Illumina) following an established metagenomics pipeline^77,78^. The infectious content of virus stocks was determined by titration on Vero E6 cells by the Spearman–Kärber method. All virus-related work was conducted in the high-containment BSL3+ facilities of the KU Leuven Rega Institute (3CAPS) under licenses AMV 30112018 SBB 219 2018 0892 and AMV 23102017 SBB 219 2017 0589 according to institutional guidelines.

Antibody VHH-72-Fc was administered i.p. at a dose of 20 mg/kg 1 day prior to infection. VHH-72-Fc was expressed in ExpiCHO cells (ThermoFisher Scientific) and purified from the culture medium as described^23^. Briefly, after transfection with pcDNA3.3-VHH-72-Fc plasmid DNA, followed by incubation at 32 °C and 5% CO_2_ for 6–7 days, the VHH-72-Fc protein in the cleared cell culture medium was captured on a 5 ml MabSelect SuRe column (GE Healthcare), eluted with a McIlvaine buffer pH 3, neutralized using a saturated Na_3_PO_4_ buffer, and buffer exchanged to storage buffer (25 mM L-Histidine, 125 mM NaCl). The antibody’s identity was verified by protein- and peptide-level mass spectrometry.

### RNA extraction and RT-qPCR

Animals were euthanized at different time points postinfection, organs were removed, and lungs were homogenized manually using a pestle and a 12-fold excess of cell culture medium (Dulbecco’s modified Eagle’s medium/2% fetal calf serum). RNA extraction was performed from homogenate of 30 mg of lung tissue with the RNeasy Mini Kit (Qiagen), or 50 µl of serum using the NucleoSpin Kit (Macherey-Nagel), according to the manufacturer’s instructions. Other organs were collected in RNALater (Qiagen) and homogenized in a bead mill (Precellys) prior to extraction. Of 100 µl eluate, 4 µl was used as template in RT-qPCR reactions. RT-qPCR was performed on a LightCycler96 platform (Roche) using the iTaq Universal Probes One-Step RT-qPCR Kit (BioRad) with primers and probes (Supplementary Table 1) specific for SARS-CoV-2, mouse β-actin (*Actb*), and hamster β-actin (*ACTB*), *IL6*, *IL10*, *IFNλ*, *IFNγ*, *MX2*, *IP-10*, *TNFα*, and *ACE2* (IDT). SARS-CoV-2 qPCR reactions were carried out in duplicate for each data point. Standards of SARS-CoV-2 cDNA (IDT) and infectious virus were used to express the amount of RNA as normalized viral genome equivalent copies per milligram tissue or as TCID_50_ equivalents per milliliter serum, respectively. The mean of housekeeping gene β-actin was used for normalization. The relative fold change was calculated using the 2^−ΔΔCt^ method^79^.

### Quantification of SARS-CoV-2 infectious particles in lung tissues

After extensive transcardial perfusion with phosphate-buffered saline (PBS), lungs were collected, extensively homogenized using manual disruption (Precellys24) in minimal essential medium (5% w/v), and centrifuged (12,000 rpm, 10 min, 4 °C) to pellet the cell debris. Infectious SARS-CoV-2 particles were quantified by means of endpoint titrations on confluent Vero E6 cell cultures. Viral titers were calculated by the Spearman–Kärber method and expressed as the TCID_50_ per 100 mg tissue.

### Differential gene expression and bioinformatics analysis

To study differential gene expression, RNA was extracted from lung tissues using Trizol, subjected to cDNA synthesis (High Capacity cDNA Reverse Transcription Kit, Thermo Fisher Scientific) and qPCR using a custom Taqman qRT-PCR array (Thermo Fisher Scientific) of 30 genes known to be activated in response to virus infection^13^, as well as two housekeeping genes (Supplementary Table 2). Data collected were analyzed using the Quant Studio Design and Analysis (version 1.5.1) and Data Assist software (version 3.01, Thermo Fisher Scientific). Pathway, Gene Ontology, and transcription factor target enrichment analysis was performed using Gene Set Enrichment Analysis (Molecular Signatures Database (MSigDB), Broad Institute). Principal component analysis, correlation matrices, and unsupervised hierarchical clustering (Euclidean distance) were performed using XLSTAT and visualized using MORPHEUS (https://software.broadinstitute.org/morpheus)^17^.

### Histology

For histological examination, the lungs were fixed overnight in 4% formaldehyde and embedded in paraffin. Tissue sections (4 µm) were stained with hematoxylin and eosin to visualize and score for lung damage.

### In vitro JAK/STAT inhibition assay

Calu-3 (human airway epithelial) cells were plated at 5 × 10^4^ cells/well in a 96-well plate and incubated overnight with 4 µM of the JAK1/2 inhibitor Ruxolitinib^80^ (Toronto Research Chemicals, ON, Canada). Next day, cells were pretreated for 4 h with 10 IU/ml of Universal type I IFN (PBL Assay Science, NJ, USA, cat. no. 11200) before infection with SARS-CoV-2 (P4 or P6, 5 × 10^3^ TCID_50_ per well). Two hours post infection, cells were washed with PBS and incubated for an additional 48 h with IFN and Ruxolitinib before collection of the supernatant for RNA extraction and quantification of virus yields by RT-qPCR.

### Micro-CT and image analysis

Hamsters were anesthetized using isoflurane (Iso-Vet) (2–3% in oxygen) and installed in prone position into the X-cube micro-CT scanner (Molecubes) using a dedicated imaging bed. Respiration was monitored throughout. A scout view was acquired and the lung was selected for a non-gated, helical CT acquisition using the High-Resolution CT protocol, with the following parameters: 50 kVp, 960 exposures, 32 ms/projection, 350 µA tube current, rotation time 120 s. Data were reconstructed using a regularized statistical (iterative) image reconstruction algorithm using non-negative least squares^81^, using an isotropic 100 µm voxel size, and scaled to Hounsfield Units after calibration against a standard air/water phantom. The spatial resolution of the reconstruction was estimated at 200 µm by minimizing the mean squared error between the three-dimensional reconstruction of the densest rod in a micro-CT multiple density rod phantom (Smart Scientific) summed in the axial direction and a digital phantom consisting of a two-dimensional disk of 17.5 mm radius that was post-smoothed with Gaussian kernels using different full width half maxima, after aligning the symmetry axis of the rod to the *z*-axis.

Visualization and quantification of reconstructed micro-CT data was performed with DataViewer and CTan software (Bruker micro-CT). As primary outcome parameter, a semi-quantitative scoring of micro-CT data was performed^33,34,82^ with minor modifications toward optimization for COVID-19 lung disease in hamsters. In brief, visual observations were scored (from 0 to 2 depending on severity, both for parenchymal and airway disease) on 5 different, predefined transversal tomographic sections throughout the entire lung image for both lung and airway disease by two independent observers (L.S. and G.V.V.) and averaged. Scores for the 5 sections were summed up to obtain a score from 0 to 10 reflecting severity of lung and airway abnormalities compared to scans of healthy, WT control hamsters. As secondary measures, image-derived biomarkers (non-aerated lung volume, aerated lung volume, total lung volume, the respective densities within these volumes, and large airway volume) were quantified as in refs. ^33,82^ for a manually delineated volume of interest in the lung, avoiding the heart and main blood vessels. The threshold used to separate the airways and aerated (gray value 0–55) from non-aerated lung volume (gray value 56–255) was set manually on an 8-bit grayscale histogram and kept constant for all data sets.

### Statistical analysis

GraphPad Prism Version 8 (GraphPad Software, Inc.) was used for all statistical evaluations. The number of animals and independent experiments that were performed is indicated in the legends to figures. Statistical significance was determined using non-parametric Mann–Whitney *U* test for pairwise comparisons or Kruskal–Wallis test with Dunn’s post hoc test for multiple comparisons. Values were considered significantly different at *P* values of ≤0.05.

### Reporting summary

Further information on research design is available in the Nature Research Reporting Summary linked to this article.

## Supplementary information

Supplementary Information

Peer Review File

Reporting Summary

## Data Availability

SARS-CoV-2 strain BetaCov/Belgium/GHB-03021/2020 sequence is available from GISAID (EPI ISL 407976|2020-02-03, https://www.gisaid.org). Prototypic Wuhan-Hu-1 2019-nCoV sequence is available from GenBank (accession number MN908947.3). All data supporting the findings in this study are also available from the corresponding author upon request. Source data are provided with this paper.

## Source data

Source Data
