# Supplementary material for: STAT2 signaling restricts viral dissemination but drives severe pneumonia in SARS-CoV-2 infected hamsters
Source: Nat Commun. 2020 Nov 17;11:5838. doi: 10.1038/s41467-020-19684-y (PMC7672082; doi:10.1038/s41467-020-19684-y)
Supplement: Supplementary file 1 — Supplementary Information [file 41467_2020_19684_MOESM1_ESM.pdf]

Phylogenetic tree showing the relationships between various SARS-CoV-2 sequences. The tree is rooted on the left and branches out to the right. The scale bar represents 0.003 substitutions per site. The sequences are color-coded by country: China (blue), USA (orange), Germany (green), France (red), and others (grey). The tree shows that the sequences are highly similar, with many branches having high Bootstrap values. The scale bar indicates a distance of 0.003 substitutions per site. The sequences are color-coded by country: China (blue), USA (orange), Germany (green), France (red), and others (grey). The tree is rooted on the left and branches out to the right. The sequences are labeled with their accession numbers and the country of origin. The tree shows that the sequences are highly similar, with many branches having high Bootstrap values. The scale bar indicates a distance of 0.003 substitutions per site.

0.003

BetaCoV/Shenzhen/HKU-SZ-005(2020)EPI_ISL_405839
 MN975262_Wuhan_seafood_market_pneumonia_virus_isolate_2019-nCoV_HKU-SZ-005b_2020
 BetaCoV/USA/AZ1(2020)EPI_ISL_406223
 MN997409_Wuhan_seafood_market_pneumonia_virus_isolate_2019-nCoV/USA-AZ1(2020)
 BetaCoV/Shenzhen/HKU-SZ-002(2020)EPI_ISL_406030
 MN938384_Wuhan_seafood_market_pneumonia_virus_isolate_2019-nCoV_HKU-SZ-002a_2020
 BetaCoV/Guangdong/GD012(2020)EPI_ISL_409932
 BetaCoV/England/01(2020)EPI_ISL_407071
BetaCoV/Belgium/GHB-03021(2020)
 BetaCoV/Wuhan/WH04(2020)EPI_ISL_406801
 BetaCoV/USA/WA1(2020)EPI_ISL_404895
 MN985325_Wuhan_seafood_market_pneumonia_virus_isolate_2019-nCoV/USA-WA1(2020)
 MN994467_Wuhan_seafood_market_pneumonia_virus_isolate_2019-nCoV/USA-CA1(2020)
 MN988713_Wuhan_seafood_market_pneumonia_virus_isolate_2019-nCoV/USA-L1(2020)
 BetaCoV/Germany/BavPat1(2020)EPI_ISL_406862
 BetaCoV/Zhejiang/WZ-01(2020)EPI_ISL_404227
 MN996527_Wuhan_seafood_market_pneumonia_virus_isolate_WIV02
 BetaCoV/Foshan/20SF707(2020)EPI_ISL_406534
 BetaCoV/Finland/1(2020)EPI_ISL_407079
 BetaCoV/Wuhan/WH03(2020)EPI_ISL_406800
 BetaCoV/Wuhan/WH01(2019)EPI_ISL_406798
 MN996528_Wuhan_seafood_market_pneumonia_virus_isolate_WIV04
 BetaCoV/China/WHU02(2020)EPI_ISL_406717
 BetaCoV/China/WHU01(2020)EPI_ISL_406716
 MN988669_Wuhan_seafood_market_pneumonia_virus_isolate_2019-nCoV_WHU02
 MN988668_Wuhan_seafood_market_pneumonia_virus_isolate_2019-nCoV_WHU01
 BetaCoV/Singapore/1(2020)EPI_ISL_406973
 BetaCoV/Hangzhou/HZ-1(2020)EPI_ISL_406970
 MN996531_Wuhan_seafood_market_pneumonia_virus_isolate_WIV07
 MN996530_Wuhan_seafood_market_pneumonia_virus_isolate_WIV05
 BetaCoV/Nonthaburi/01(2020)EPI_ISL_403962
 MN996529_Wuhan_seafood_market_pneumonia_virus_isolate_WIV05
 BetaCoV/Wuhan-Hu-12019(EPI_ISL_400125)
 MN990647_3_Wuhan_seafood_market_pneumonia_virus_isolate_Wuhan-Hu-1
 NC_045512.2_Wuhan_seafood_market_pneumonia_virus_isolate_Wuhan-Hu-1
 BetaCoV/Australia/VIC01(2020)EPI_ISL_406844
 BetaCoV/Taiwan/2(2020)EPI_ISL_406031
 BetaCoV/France/IDF0372(2020)EPI_ISL_406596
 MN994468_Wuhan_seafood_market_pneumonia_virus_isolate_2019-nCoV/USA-CA2(2020)

SARS-CoV-2 strain BetaCoV/Belgium/GHB-03021/2020 (direct sequencing)
from nasal swap obtained from traveler returning from Wuhan in early 02/2020

Passage (P)<br>Cell line<br>Trypsin (4 µg/mL)<br>Antibiotics/fungicide	P1<br>HuH7<br>yes<br>yes	P2<br>Vero-E6<br>yes<br>yes	P3<br>Vero-E6<br>no<br>no	P4<br>Vero-E6<br>no<br>yes	P5<br>Vero-E6<br>no<br>no	P6<br>Vero-E6<br>no<br>no
<i>Exclusion of adventitious agents<br>(clinical microbiology panel)</i>						
Days in culture<br>Cytopathic effect<br>Titer (TCID ₅₀ /mL)	7<br>50%<br>-	7<br>80-90%<br>-	5<br>80-90%<br>-	5<br>full<br>3.8x10 ⁶	3<br>full<br>3.3x10 ⁴	3<br>full<br>4.8x10 ⁷
Sequencing<br>**S protein variants**	Minion<br>**Mixed population**<br>85% WT +<br>15% 9+5aa del				Minion<br>100% 9+<br>5aa del	

50
 1
 Wuhan-Hu-1 STQDLFLPFFSNVTWFHAIHVSGTNGTKRFDNPVLPFNDGV
 GHB-P0 STQDLFLPFFSNVTWFHAIHVSGTNGTKRFDNPVLPFNDGV
 GHB-P6 STQDLFLPFFSNVTWFHA-----KRFDNVLPFNDGV
 SARS-CoV-1 (Tor2) LTQDLFLPFYSNVTWFHT-----HTFGNPVLPFKDGI
 ***** ** *

658
 1
 Wuhan-Hu-1 NSYECDIPIGAGICAS^YQTQ^TNS^PRR^RRS^SVASQSI^IIAYTMS
 GHB-P0 NSYECDIPIGAGICAS^YQTQ^TNS^PRR^RRS^SVASQSI^IIAYTMS
 GHB-P6 NSYECDIPIGAGICAS^YQ-----P^RRR^RRS^SVASQSI^IIAYTMS
 SARS-CoV-1 (Tor2) TSYECDIPIGAGICAS^YHTVSL^L-----R^TSS^SQKSIVAYTMS
 ***** * *

S1/2

Fig. S1. SARS-CoV-2 phylogeny and stock generation. (a) Phylogenetic tree of recent clinical SARS-CoV-2 isolates, with the SARS-CoV-2 strain used in the current study indicated in red. Phylogenetic analysis confirmed a close evolutionary relationship of the isolate BetaCoV/Belgium/GHB-03021/2020 (EPI ISL 407976|2020-02-03) with the prototypic Wuhan-Hu-1 2019-nCoV strain (GenBank accession number MN908947.3). **(b)** Passaging (P) history of the SARS-CoV-2 isolate used indicating the origin and key characteristics of the virus stocks P4 and P6. Two in-frame deletions in the N-terminal domain and the furin-cleavage site of Spike (S) glycoprotein (9aa and 5aa, respectively) were observed in P4 (mixed population of 85% WT genomes and 15% (9+5aa del) mutant genomes). P6 only contains the (9+5aa del) mutant genomes. As a consequence, P4 is an intermediate between the original P0 WT virus and the P6 virus. **(c)** Alignment of parts of the Spike protein of SARS-CoV-2 isolates P0 and P6 with those of the prototypic Wuhan-Hu-1 strain^{1,2}, and SARS-CoV-1 (Tor2 strain)³. Upper panel – deletion discovered in the N-terminal domain (NTD); lower panel – deletion at the polybasic furin recognition site (shaded box)^{4,5}; * – residue conserved between SARS-CoV-1 and SARS-CoV-2; **BOLD** – predicted O-glycosylation acceptor⁶; S1/S2 – junction between the membrane-distal S1 and membrane-proximal S2 subunits.

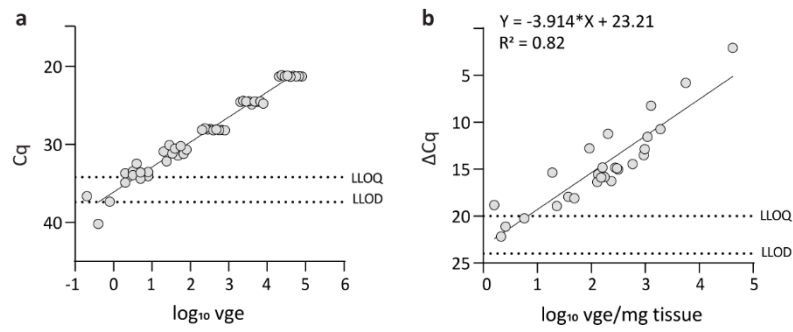

Fig. S2. Correlation curves for quantification of viral loads in mice. (a) Correlation between cycle of quantification (Cq) and log₁₀ viral genome equivalents (vge) obtained from a cDNA plasmid standard. **(b)** Correlation between delta Cq and log₁₀ vge/mg lung tissue in mice. Delta Cq values are calculated by subtracting Cq values of β-actin from Cq values of SARS-CoV-2. To express viral loads in the lungs of mice, this correlation was used to transform delta Cq values into *Normalized log₁₀ vge/mg tissue*. Lowest limit of quantification (LLOQ) was defined as lowest detectable value in the linear range of the calibration curve. Lowest limit of detection (LLOD) was defined as lowest detectable value below the linear range of the calibration curve.

a Lung pathology H&E scoring

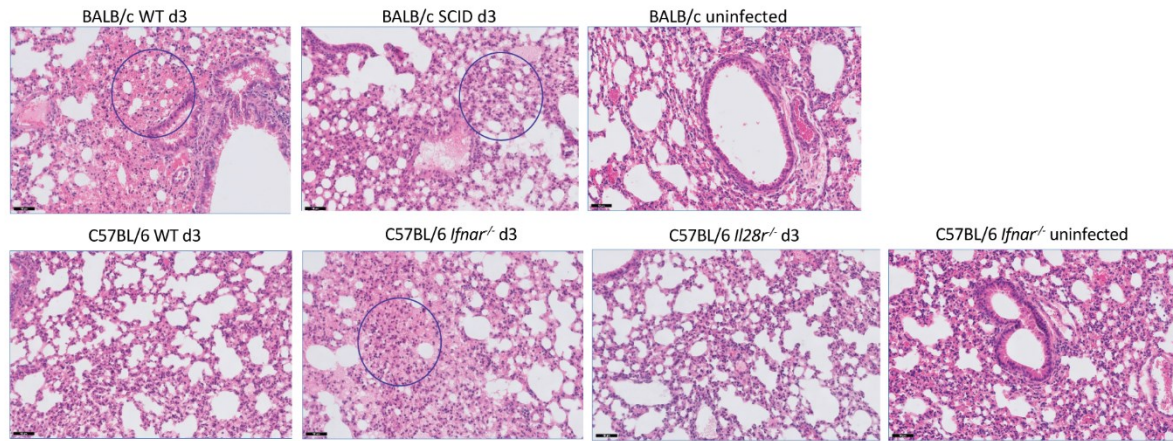

b Histopathological scores of H&E images

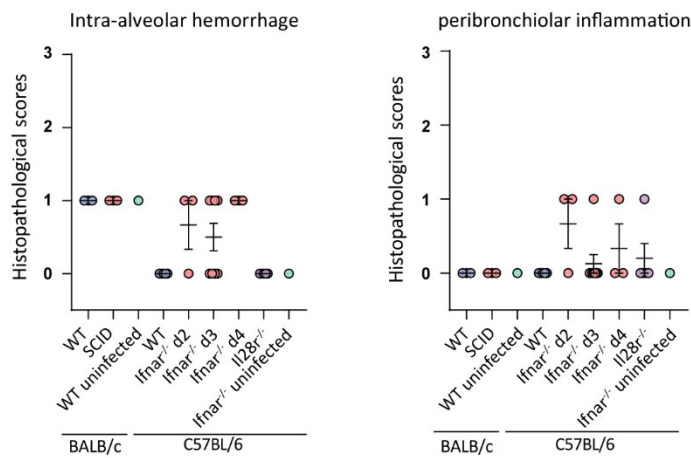

Fig. S3. Lung pathology in mice. (a) Representative H&E images of the lungs of uninfected (green circles, n=1) or SARS-CoV-2 infected WT (blue circles, n=3) and SCID (red circles, n=3) BALB/c and WT (blue circles, n=5), *Ifnar1*^{-/-} (red circles; day 2 d.p.i. (n=3), day 3 d.p.i. (n=8), day 4 d.p.i. (n=3)) and *Il28r*^{-/-} (purple circles, n=5) C57BL/6 mice. Circles indicate presence of intra-alveolar hemorrhage. Black scalebar: 50µm **(b)** Histopathological scoring on a scale of 0-3 of intra-alveolar hemorrhage and peribronchiolar inflammation (WT – blue circles; SCID or *Ifnar1*^{-/-} – red; *Il28r*^{-/-} – purple; uninfected controls – green). The data shown are means ± SEM.

Hierarchical clustering analysis

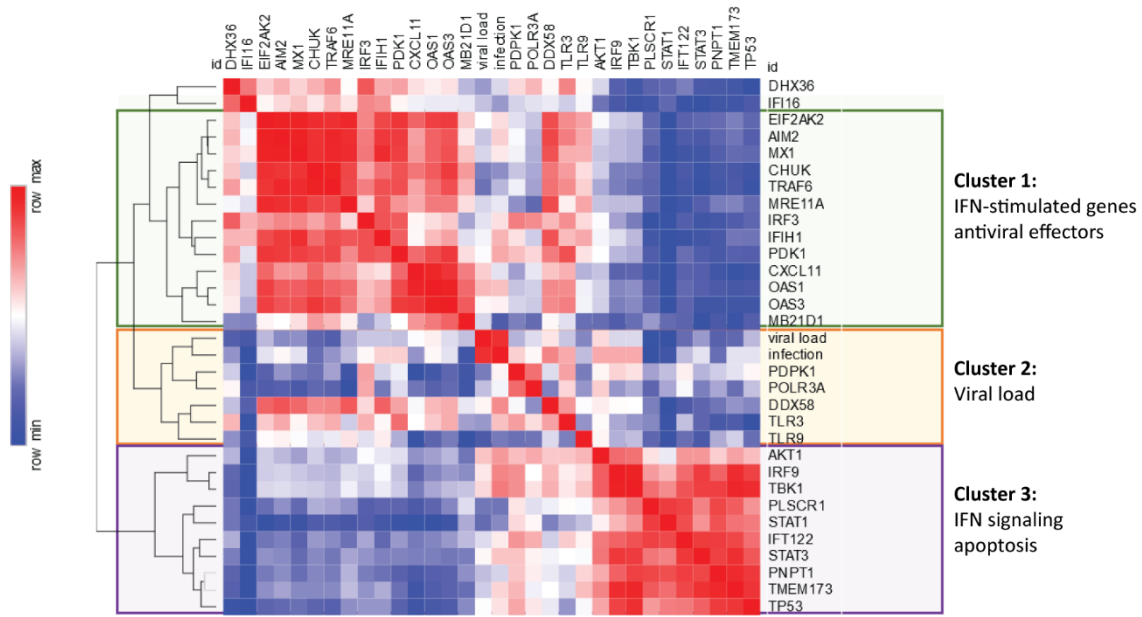

Fig. S4. Hierarchical clustering analysis of transcriptomic data from uninfected and SARS-CoV-2-infected *Ifnar1*^{-/-} mice. Cluster dendrogram (Euclidean distance) and heatmap of Spearman correlation coefficients between molecular and virological data from uninfected and SARS-CoV-2-infected *Ifnar1*^{-/-} mice (n=3 each, analysis on samples of day 3 p.i.), based on a transcriptomic analysis of 30 selected marker genes⁷ (Table S2). Hierarchical cluster analysis reveals three main clusters as indicated. A small intermediate cluster (Cluster 2) containing nucleic acid sensors *TLR3/TLR9/DDX58/POLR3A* correlates with viral load. Clusters 1 and 3 are negatively correlated to each other and independent of viral load. Cluster 1 is enriched (p<0.001) for antiviral effector molecules such as *cGAS*, *Mx1*, *IFIH1/MDA-5*, *IRF3*, *OAS1*, *OAS3* and *PKR/EIF2AK2*⁸. Cluster 3 comprises upstream regulators *STAT1*, *STAT3* and *STING/TMEM173* but also *PLSCR1* (scramblase) and *p53*, linking IFN signaling to apoptosis (enrichment p<0.001)⁹. In contrast, *IFI16* decreases upon SARS-CoV-2 infection and clusters independently with *DHX36*, suggesting absence of inflammatory pyroptosis and/or *DHX36*-amplified innate immunity, in line with a fast control of viral replication and limited tissue damage as observed in mice¹⁰.

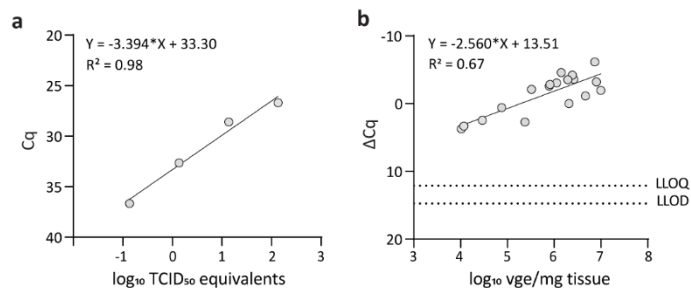

Fig. S5. Correlation curves for quantification of viral loads in hamsters. (a) Correlation between cycle of quantification (Cq) and log₁₀ TCID₅₀ equivalents obtained from an infectious virus standard. **(b)** Correlation between delta Cq and log₁₀ vge/mg tissue in hamsters. Delta Cq values are calculated by subtracting Cq values of β-actin from Cq values of SARS-CoV-2. To express viral loads in the different tissue of hamsters, this correlation was used to transform delta Cq values into *Normalized log₁₀ vge/mg tissue*. Lowest limit of quantification (LLOQ) was defined as lowest detectable value in the linear range of the calibration curve. Lowest limit of detection (LLOD) was defined as lowest detectable value below the linear range of the calibration curve.

Viral RNA in human Calu-3 cells

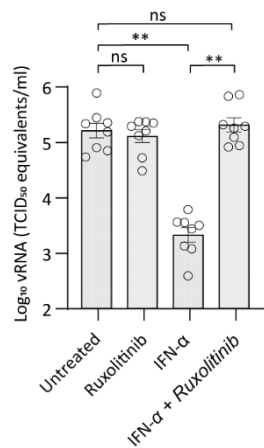

Fig. S6. Inhibition of JAK/STAT signaling by Ruxolitinib can rescue SARS-CoV-2 virus replication from the antiviral effect of Type-I IFN. Calu-3 cells¹¹ were left untreated or treated with Ruxolitinib (4 μ M), IFN- α (10 IU/mL) or a combination of both (n=8 for each condition). Treatment was initiated 4 h before infection and was continued through the whole experiment. Cultures were infected with P4 SARS-CoV-2 (MOI of 0.1), 48 h p.i. cell culture SN was collected, RNA was extracted and vRNA levels were quantified using RT-qPCR. The data shown are means $\pm$ SEM. Statistical significance between groups was calculated by Kruskal-Wallis with a two-sided Dunn's post hoc test. P values: **P = 0.0035 and **P=0.0019 (left to right); ns, not significant.

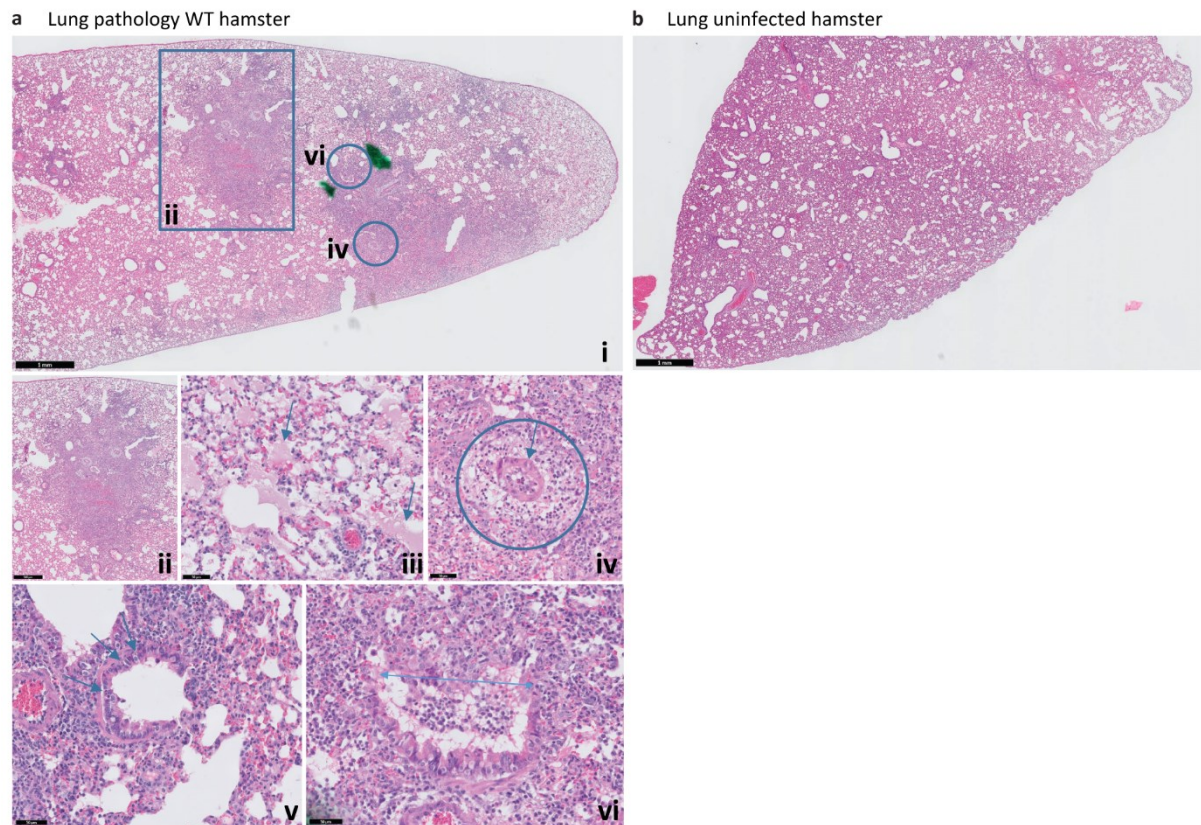

Fig. S7. Lung pathology in a WT hamster. **(a)** Representative H&E images of the lung of a SARS-CoV-2 infected WT hamster. **(Panel i)** rectangle bronchopneumonia (see also panel b), upper circle necrotizing bronchiolitis (see also panel f), lower circle perivascular edema (see also panel d) (black scalebar: 1mm). **(Panel ii)** area of bronchopneumonia with inflammation centered around a bronchus (magnification of panel a) (black scalebar: 0.5mm). **(Panel iii)** intra-alveolar hemorrhage and edema (arrows) (black scalebar: 50 μ m). **(Panel iv)** perivascular edema (circle), vascular wall (arrow) is surrounded by loose fibrous tissue and a few inflammatory cells (magnification of panel a) (black scalebar: 50 μ m). **(Panel v)** bronchus with apoptotic cells (arrows) (black scalebar: 50 μ m). **(Panel vi)** necrotizing bronchiolitis: half of the bronchus wall has disappeared and is replaced by inflammatory cells (arrow, magnification of panel a) (black scalebar: 50 μ m). **(b)** Representative H&E image of the lung of an uninfected WT hamster (black scalebar: 1mm). The images in **a** are representative images from two independent experiments (n=7 animals); the image in **b** is from one experiment (n=1 animal)

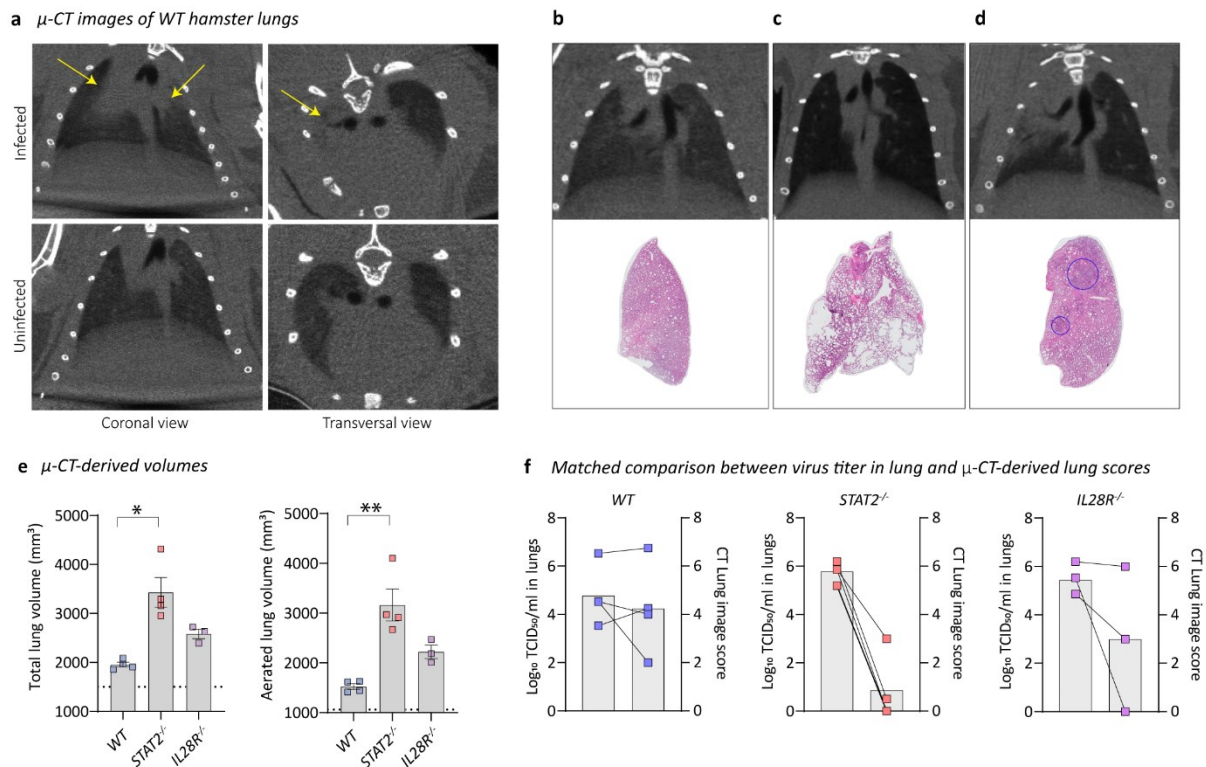

Fig. S8. Micro-CT-observed lung pathology. (a) Representative coronal and transversal micro-CT (μ CT) images of an upper airway enlargement and dense lung infiltrates in the lungs of infected (P6 SARS-CoV-2) WT hamsters compared to uninfected hamsters. Yellow arrows indicate examples of pulmonary infiltrates seen as consolidation of lung parenchyma. (b) Coronal micro-CT image (left top) and histopathological H&E-stained section of whole lobe (left bottom) and of a healthy, uninfected hamster lung. (c) Histopathology confirmed hyperinflation visualized on the micro-CT scan as decreased density (blackening) of lung tissue and overall lung enlargement, seen in one infected *STAT2*^{-/-} hamster. (d) Consolidation of right lung parenchyma on micro-CT corresponds to bronchopneumonia evidenced by histopathology (darker blue stained areas, blue circles) of an infected *IL28R*^{-/-} hamster. (e) Micro-CT-derived biomarkers quantifying total lung volume and aerated lung volume (see also Fig. 3e) of infected WT (blue squares, n=4), *STAT2*^{-/-} (red squares, n=4) and *IL28R*^{-/-} (purple squares, n=3) hamsters. Lines indicate micro-CT-derived biomarkers in healthy animals (n=3). The data shown are means $\pm$ SEM. (f) Matched comparison between infectious viral load in the lung (left Y-axis) (values from Fig. 2c) and μ CT-derived semi-quantitative lung scores (values from Fig. 3d) (right Y-axis) (n=4 for WT and *STAT2*^{-/-} and n=3 for *IL28R*^{-/-}). Lines indicate matched samples. Statistical significance between genotypes was calculated by the Kruskal-Wallis with a two-sided Dunn's post hoc test. P values: *P=0.043 and *P=0.0030 (left to right in e).

a Gene expression in lung

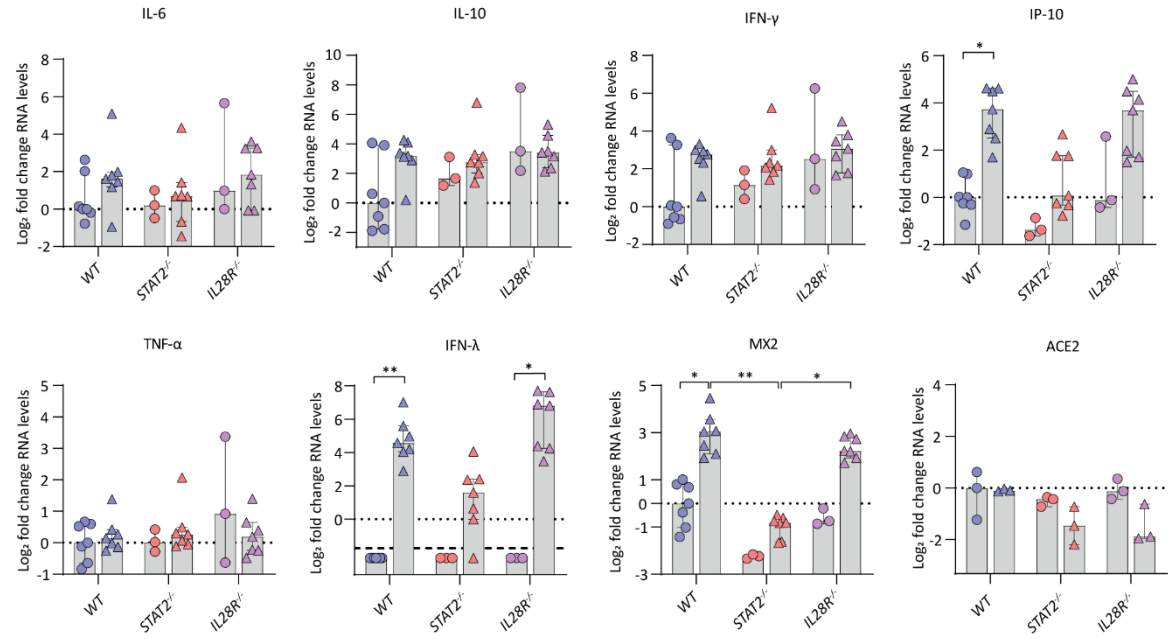

b Spearman Correlations

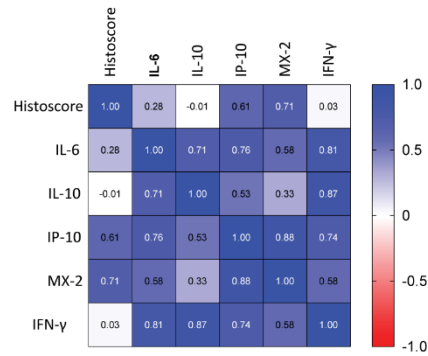

Fig. S9. RNA expression levels after SARS-CoV-2 infection. (a) Individual expression profiles for IL-6, IL-10, IFN-γ, IP-10, TNF-α, IFN-λ, MX2 and ACE2 in lungs of WT (blue), *STAT2*^{-/-} (red) and *IL28R*^{-/-} (purple) hamsters four days after SARS-CoV-2 infection (n=7 for each genotype) (as in Figure 3f and g) presented as log₂-fold change relative to uninfected WT controls (blue circles, n=7). Levels of individual mRNAs in uninfected (circles) and infected (triangles) animals were determined by RT-qPCR and normalized for β-actin mRNA. Changes are reported as values over the median of uninfected controls (dotted line) calculated using the $2^{(-\Delta\Delta Cq)}$ method. Only for IFN-λ, where control animals had undetectable mRNA levels, values were compared to the lowest detectable value (dashed line). Data presented as median ± IQR. **(b)** Heatmap of Spearman correlation coefficients between normalized gene expression levels (Fig. 3f and panel a of this figure) and histology data (derived from Fig. 3a) from uninfected and SARS-CoV-2-infected hamsters. Positive correlations were used in Fig. 3h to show a correlation between increased lung pathology and increased gene expression in the different genotypes. Statistical significance between groups was calculated by Kruskal-Wallis with a two-sided Dunn's post hoc test. P values: *P=0.012 (top a); **P=0.0061, *P=0.026, *P=0.039, **P=0.0014 and *P=0.012 (bottom left to right a).

Supplementary Table S1. Primers and probes used for RT-qPCR

Gene	Description	Oligonucleotide sequence
**SARS-CoV-2**	Primer 1	5'-TTA CAA ACA TTG GCC GCA AA-3'
	Primer 2	5'-GCG CGA CAT TCC GAA GAA-3'
	Probe	5'-FAM-ACA ATT TGC CCC CAG CGC TTC AG-BHQ1-3'
**Mouse <i>Actb</i>**	Primer 1	5'-GAT TAC TGC TCT GGC TCC TAG-3'
	Primer 2	5'-GAC TCA TCG TAC TCC TGC TTG-3'
	Probe	5'-FAM-CTG GCC TCA CTG TCC ACC TTC C-ZEN/IABkFQ-3'
**Hamster <i>ACE2</i>**	Primer 1	5'-GGG AAC TGT CAA AGG GTA CAG-3'
	Primer 2	5'-CCC TTC CTA CAT CAG TCC TAC T-3'
	Probe	5'-FAM-TCC CTG CTC ATT TGC TTG GTG ACA-ZEN/IABkFQ-3'
**Hamster <i>ACTB</i>**	Primer 1	5'-GGC CAG GTC ATC ACC ATT-3'
	Primer 2	5'-GAG TTG AAT GTA GTT TCG TGG ATG-3'
	Probe	5'-Cy5-TTT CCA GCC TTC CTT CCT GGG TAT G-IBRQ-3'
**Hamster <i>IFN-γ</i>**	Primer 1	5'-TTT CTC CAT GCT GCT GTT GAA-3'
	Primer 2	5'-GGC CAT CCA GAG GAG CAT AG-3'
	Probe	5'-FAM-CAC CAT CAA GGC AGA CCT GTT TGC TAA CTT-ZEN/IABkFQ-3'
**Hamster <i>IFNλ</i>**	Primer 1	5'-CCC ACC AGA TGC AAA GGA TT-3'
	Primer 2	5'-CTT GAG CAG CCA CTC TTC TAT G-3'
	Probe	5'-FAM-ACA TAG CCC GGT TCA AGT CTC TGC-ZEN/IABkFQ-3'
**Hamster <i>IL-6</i>**	Primer 1	5'-GGT ATG CTA AGG CAC AGC ACA CT-3'
	Primer 2	5'-CCT GAA AGC ACT TGA AGA ATT CC-3'
	Probe	5'-FAM-AGA AGT CAC CAT GAG GTC TAC TCG GCA AAA-ZEN/IABkFQ-3'
**Hamster <i>IL-10</i>**	Primer 1	5'-TTC TGG CCC GTG GTT CTC T-3'
	Primer 2	5'-GTT GCC AAA CCT TAT CAG AAA TGA-3'
	Probe	5'-FAM-CAG TTT TAC CTG GTA GAA GTG ATG CCC CAG G-ZEN/IABkFQ-3'
**Hamster <i>IP-10</i>**	Primer 1	5'-GCC ATT CAT CCA CAG TTG ACA-3'
	Primer 2	5'-CAT GGT GCT GAC AGT GGA GTC T-3'
	Probe	5'-FAM-CGT CCC GAG CCA GCC AAC GA-ZEN/IABkFQ-3'
**Hamster <i>MX2</i>**	Primer 1	5'-CCA GTA ATG TGG ACA TTG CC-3'
	Primer 2	5'-CAT CAA CGA CCT TGT CTT CAG TA-3'
	Probe	5'-FAM-TGT CCA CCA GAT CAG GCT TGG TCA-ZEN/IABkFQ-3'
**Hamster <i>TNF-α</i>**	Primer 1	5'-AGC TGG TTG TCT TTG AGA GAC ATG-3'
	Primer 2	5'-GGA GTG GCT GAG CCA TCG T-3'
	Probe	5'-FAM-CCA ATG CCC TCC TGG CCA ACG-ZEN/IABkFQ-3'

Supplementary Table S2. RT-qPCR Taqman assays for analysis of DEG in SARS-CoV-2 infected cells

Gene of Interest	Proposed MoA in immune response	Reference	Assay ID
<i>18s rRNA</i>	House keeping gene		Hs99999901_s1
<i>GAPDH</i>	House keeping gene		Mm99999915_g1
<i>AIM2</i>	Cytosolic DNA sensor	Yu and Levine ¹²	Mm01295719_m1
<i>AKT1</i>	Controls innate immune cell development and function	Zhang et al ¹³	Mm01331626_m1
<i>CHUK (IKK)</i>	Inhibitor of nuclear factor kappa-B kinase subunit alpha (IKK- α)	Llona-Minguez et al ¹⁴	Mm00432529_m1
<i>CXCL11</i>	Interferon inducible T cell alpha chemoattractant	Venter et al ¹⁵	Mm00444662_m1
<i>DHX36</i>	Enhances RIG-I Signaling	Yoo et al ¹⁶	Mm00661002_m1
<i>DDX58</i>	Antiviral, IFN signalling, innate immune receptor	Pulendran et al ¹⁷ , Querec et al ¹⁸	Mm01216853_m1
<i>EIF2AK 2</i>	Antiviral, IFN signalling	Querec et al ¹⁸ , Gaucher et al ¹⁹	Mm01235643_m1
<i>IFI16</i>	Interferon Gamma Inducible Protein 16	Trapani et al ²⁰	Mm00492602_m1
<i>IFIH1</i>	Antiviral, IFN signalling, innate immune receptor	Pulendran et al ²¹ , Querec et al ²²	Mm00459183_m1
<i>IFT122</i>	Intraflagellar Transport 122	Boubakri et al ²³	Mm00661643_m1
<i>IRF3</i>	Interferon regulatory factor 3	Collins et al ²⁴	Mm00516784_m1
<i>IRF9</i>	Antiviral, IFN signalling	Gaucher et al ¹⁹	Mm00492679_m1
<i>MB21D1(cGAS)</i>	Cytosolic DNA sensor	Liang et al ²⁵	Mm01147496_m1
<i>MRE11</i>	Double strand break repair nuclease	Shibata et al ²⁶	Mm00450600_m1
<i>OAS1</i>	Antiviral, IFN signalling	Gaucher et al ¹⁹	Mm00449297_m1
<i>OAS3</i>	Antiviral, IFN signalling	Gaucher et al ¹⁹	Mm00460944_m1
<i>MX1</i>	Antiviral, IFN signalling	Querec et al ¹⁸ , Gaucher et al ¹⁹	Mm00487796_m1
<i>PDK1</i>	Key role in regulation of glucose and fatty acid metabolism	Tan et al ²⁷	Mm00554300_m1
<i>PDPK1</i>	Important role in the signalling pathways	Sato et al ²⁸	Mm00440707_m1
<i>PLSCR1</i>	Antiviral, IFN signalling	Querec et al ¹⁸ , Gaucher et al ¹⁹	Mm01228223_g1
<i>PNPT1</i>	Antiviral, IFN signalling, innate immune receptor	Pulendran et al ²¹ , Querec et al ¹⁸	Mm00466286_m1

<i>STAT1</i>	Key regulators of the early innate immune response	Decker et al ²⁹	Mm01257286_m1
<i>STAT3</i>	Control of inflammation and immunity	Hillmer et al ³⁰	Mm01219775_m1
<i>TBK1</i>	Protein kinase downstream of TMEM173 (STING)	Lam et al ³¹	Mm00451150_m1
<i>TLR3</i>	pattern-recognition receptors (PRRs)	Yu and Levine ¹²	Mm01207404_m1
<i>TLR9</i>	Cytosolic DNA sensor	Yu and Levine ¹²	Mm07299609_m1
<i>TMEM173(STING)</i>	Stimulator of IFN response	Lam et al ³¹	Mm01158117_m1
<i>TP53</i>	Tumor protein 53	Matlashewski et al ³²	Mm00480750_m1
<i>TRAF6</i>	TNF receptor associated factor (TRAF)	Youseff et al ³³	Mm00493836_m1

References to Supplementary Material

1. Wrapp, D. *et al.* Cryo-EM structure of the 2019-nCoV spike in the prefusion conformation. *Science* (80-.). **367**, 1260–1263 (2020).
2. Wu, F. *et al.* A new coronavirus associated with human respiratory disease in China. *Nature* **579**, 265–269 (2020).
3. Marra, M. A. *et al.* The genome sequence of the SARS-associated coronavirus. *Science* (80-.). **300**, 1399–1404 (2003).
4. Zhang, T., Wu, Q. & Zhang, Z. Probable Pangolin Origin of SARS-CoV-2 Associated with the COVID-19 Outbreak. *Curr. Biol.* **30**, 1346-1351.e2 (2020).
5. Zhang, Y.-Z. & Holmes, E. C. A Genomic Perspective on the Origin and Emergence of SARS-CoV-2. *Cell* (2020) doi:10.1016/j.cell.2020.03.035.
6. Andersen, K. G., Rambaut, A., Lipkin, W. I., Holmes, E. C. & Garry, R. F. The proximal origin of SARS-CoV-2. *Nat. Med.* **26**, 450–452 (2020).
7. Sharma, S. *et al.* Small molecule inhibitors of TBK1 serve as adjuvant for a plasmid-launched live-attenuated yellow fever vaccine. *Hum. Vaccines Immunother.* (2020).
8. Schoggins, J. W. Interferon-Stimulated Genes: What Do They All Do? *Annu. Rev. Virol.* **6**, 567–584 (2019).
9. Khouri, R. *et al.* A genetic IFN/STAT1/FAS axis determines CD4 T stem cell memory levels and apoptosis in healthy controls and Adult T-cell Leukemia patients. *Oncoimmunology* **7**, (2018).
10. Briard, B., Place, D. E. & Kanneganti, T. D. DNA sensing in the innate immune response. *Physiology* vol. 35 112–124 (2020).
11. Hoffmann, M. *et al.* SARS-CoV-2 Cell Entry Depends on ACE2 and TMPRSS2 and Is Blocked by a Clinically Proven Protease Inhibitor. *Cell* **181**, 271-280.e8 (2020).
12. Yu, M. & Levine, S. J. Toll-like receptor 3, RIG-I-like receptors and the NLRP3 inflammasome: Key modulators of innate immune responses to double-stranded RNA viruses. *Cytokine and Growth Factor Reviews* vol. 22 63–72 (2011).
13. Zhang, Y. *et al.* Kinase AKT controls innate immune cell development and function. *Immunology* **140**, 143–152 (2013).
14. Llona-Minguez, S., Baiget, J. & Mackay, S. P. Small-molecule inhibitors of I κ B kinase (IKK) and IKK-related kinases. *Pharm. Pat. Anal.* **2**, 481–498 (2013).
15. Venter, M. *et al.* Gene expression in mice infected with West Nile virus strains of different neurovirulence. *Virology* **342**, 119–140 (2005).
16. Yoo, J. S. *et al.* DHX36 Enhances RIG-I Signaling by Facilitating PKR-Mediated Antiviral Stress Granule Formation. *PLoS Pathog.* **10**, (2014).
17. Pulendran, B. Learning immunology from the yellow fever vaccine: Innate immunity to systems vaccinology. *Nature Reviews Immunology* vol. 9 741–747 (2009).
18. Querec, T., Akondy, R., Lee, E., ... W. C.-N. & 2009, undefined. Systems biology approach predicts immunogenicity of the yellow fever vaccine in humans. *nature.com*.
19. Gaucher, D. *et al.* Yellow fever vaccine induces integrated multilineage and polyfunctional immune responses. *J. Exp. Med.* (2008) doi:10.1084/jem.20082292.

20. Trapani, J. A., Dawson, M., Apostolidis, V. A. & Browne, K. A. Genomic organization of IFI16, an interferon-inducible gene whose expression is associated with human myeloid cell differentiation: correlation of predicted protein domains with exon organization. *Immunogenetics* **40**, 415–424 (1994).
21. Pulendran, B. Learning immunology from the yellow fever vaccine: Innate immunity to systems vaccinology. *Nature Reviews Immunology* vol. 9 741–747 (2009).
22. Querec, T. D. *et al.* Systems biology approach predicts immunogenicity of the yellow fever vaccine in humans. *Nat. Immunol.* **10**, 116–125 (2009).
23. Boubakri, M. *et al.* Loss of ift122, a Retrograde Intraflagellar Transport (IFT) complex component, leads to slow, progressive photoreceptor degeneration due to inefficient opsin transport. *J. Biol. Chem.* **291**, 24465–24474 (2016).
24. Collins, S. E., Noyce, R. S. & Mossman, K. L. Innate Cellular Response to Virus Particle Entry Requires IRF3 but Not Virus Replication. *J. Virol.* **78**, 1706–1717 (2004).
25. Liang, Y. & Peng, H. STING-cytosolic DNA sensing: The backbone for an effective tumor radiation therapy. *Annals of Translational Medicine* vol. 4 (2016).
26. Shibata, A. *et al.* DNA Double-Strand Break Repair Pathway Choice Is Directed by Distinct MRE11 Nuclease Activities. *Mol. Cell* **53**, 7–18 (2014).
27. Tan, J. *et al.* PDK1 signaling toward PLK1-MYC activation confers oncogenic transformation, tumor-initiating cell activation, and resistance to mTOR-targeted therapy. *Cancer Discov.* **3**, 1156–1171 (2013).
28. Sato, S., Fujita, N. & Tsuruo, T. Regulation of kinase activity of 3-phosphoinositide-dependent protein kinase-1 by binding to 14-3-3. *J. Biol. Chem.* **277**, 39360–39367 (2002).
29. Decker, T., Stockinger, S., Karaghiosoff, M., Müller, M. & Kovarik, P. IFNs and STATs in innate immunity to microorganisms. *J. Clin. Invest.* **109**, 1271–1277 (2002).
30. Hillmer, E. J., Zhang, H., Li, H. S. & Watowich, S. S. STAT3 signaling in immunity. *Cytokine and Growth Factor Reviews* vol. 31 1–15 (2016).
31. Lam, E., Stein, S. & Falck-Pedersen, E. Adenovirus Detection by the cGAS/STING/TBK1 DNA Sensing Cascade. *J. Virol.* **88**, 974–981 (2014).
32. Matlashewski, G. *et al.* Isolation and characterization of a human p53 cDNA clone: expression of the human p53 gene. *EMBO J.* **3**, 3257–3262 (1984).
33. Youseff, B. H. *et al.* TRAF6 Plays a Proviral Role in Tick-Borne Flavivirus Infection through Interaction with the NS3 Protease. *iScience* **15**, 489–501 (2019).
