# Supplementary material for: STAT2 signaling restricts viral dissemination but drives severe pneumonia in SARS-CoV-2 infected hamsters
Source: Nat Commun. 2020 Nov 17;11:5838. doi: 10.1038/s41467-020-19684-y (PMC7672082; doi:10.1038/s41467-020-19684-y)
Supplement: Supplementary file 3 — Reporting Summary [file 41467_2020_19684_MOESM3_ESM.pdf]

nature research

Corresponding author(s): Hendrik Jan Thibaut, Johan Neyts and Kai Dallmeier

Last updated by author(s): Oct 8, 2020

Reporting Summary

Nature Research wishes to improve the reproducibility of the work that we publish. This form provides structure for consistency and transparency in reporting. For further information on Nature Research policies, see Authors & Referees and the Editorial Policy Checklist .

Statistics

For all statistical analyses, confirm that the following items are present in the figure legend, table legend, main text, or Methods section.

n/a	Confirmed	
☐	☒	The exact sample size (*n*) for each experimental group/condition, given as a discrete number and unit of measurement
☐	☒	A statement on whether measurements were taken from distinct samples or whether the same sample was measured repeatedly
☐	☒	The statistical test(s) used AND whether they are one- or two-sided<br>*Only common tests should be described solely by name; describe more complex techniques in the Methods section.*
☐	☒	A description of all covariates tested
☐	☒	A description of any assumptions or corrections, such as tests of normality and adjustment for multiple comparisons
☐	☒	A full description of the statistical parameters including central tendency (e.g. means) or other basic estimates (e.g. regression coefficient) AND variation (e.g. standard deviation) or associated estimates of uncertainty (e.g. confidence intervals)
☐	☒	For null hypothesis testing, the test statistic (e.g. *F*, *t*, *r*) with confidence intervals, effect sizes, degrees of freedom and *P* value noted<br>*Give P values as exact values whenever suitable.*
☒	☐	For Bayesian analysis, information on the choice of priors and Markov chain Monte Carlo settings
☒	☐	For hierarchical and complex designs, identification of the appropriate level for tests and full reporting of outcomes
☒	☐	Estimates of effect sizes (e.g. Cohen's *d*, Pearson's *r*), indicating how they were calculated

Our web collection on statistics for biologists contains articles on many of the points above.

Software and code

Policy information about availability of computer code

Data collection	Deep sequencing was performed on a MiSeq platform (Illumina).<br>Visualization and quantification of reconstructed micro-CT data was performed with DataViewer and CTan software (Version 1.20.3.0 Bruker micro-CT).
Data analysis	Data collected were analysed using the Quant Studio Design and Analysis (version 1.5.1) and Data Assist software (version 3.01, Thermo Fischer Scientific). Pathway, GO (Gene Ontology) and transcription factor target enrichment analysis was performed using GSEA (Gene Set Enrichment Analysis, Molecular Signatures Database (MSigDB), Broad Institute). Principal component analysis, correlation matrices, unsupervised hierarchical clustering (Eucledian distance) were performed using XLSTAT (Version 2020.3.1) and visualized using MORPHEUS (https://software.broadinstitute.org/morpheus).<br>GraphPad Prism Version 8 (GraphPad Software, Inc.) was used for all statistical evaluations.

For manuscripts utilizing custom algorithms or software that are central to the research but not yet described in published literature, software must be made available to editors/reviewers. We strongly encourage code deposition in a community repository (e.g. GitHub). See the Nature Research guidelines for submitting code & software for further information.

Data

Policy information about availability of data

All manuscripts must include a data availability statement. This statement should provide the following information, where applicable:

- Accession codes, unique identifiers, or web links for publicly available datasets
- A list of figures that have associated raw data
- A description of any restrictions on data availability

SARS-CoV-2 strain BetaCov/Belgium/GHB-03021/2020 sequence available from GISAID (EPI ISL 407976|2020-02-03, https://www.gisaid.org). Prototypic Wuhan-Hu-1 2019-nCoV sequence is available from GenBank (accession number MN908947.3). Source data are provided with this paper. All data supporting the findings in this study are also available from the corresponding author upon request.

Field-specific reporting

Please select the one below that is the best fit for your research. If you are not sure, read the appropriate sections before making your selection.

- [x] Life sciences
- [ ] Behavioural & social sciences
- [ ] Ecological, evolutionary & environmental sciences

For a reference copy of the document with all sections, see nature.com/documents/nr-reporting-summary-flat.pdf

Life sciences study design

All studies must disclose on these points even when the disclosure is negative.

Sample size	Sample sizes were chosen as a balance between (i) the sample size to equal or exceed what has been established in other comparable studies (e.g. PMID: 32215622; PMID: 32408338), (ii) capacity of the high-containment facilities, and (iii) limited availability of animals, in particular from the KO hamster breeds; instead of being predetermined using statistical methods to estimate power ex ante. Pivotal studies have been performed in independent biological repeats.
Data exclusions	Sub-par resolution of a CT-scan of one IL28R-/- hamster resulted in its exclusion from analysis.
Replication	Principal data could be successfully replicated in duplicate experiments. In case of systematical variation between replicates, data points from each experiment received unique symbol shapes.
Randomization	Animals were used upon availability or picked randomly in case of sufficient availability.
Blinding	Data acquisition/analysis of RT-qPCR, CT scans, virus titrations, histology and zymography was performed blinded.

Reporting for specific materials, systems and methods

We require information from authors about some types of materials, experimental systems and methods used in many studies. Here, indicate whether each material, system or method listed is relevant to your study. If you are not sure if a list item applies to your research, read the appropriate section before selecting a response.

Materials & experimental systems

n/a	Involved in the study	
x		Antibodies
	x	Eukaryotic cell lines
x		Palaeontology
	x	Animals and other organisms
x		Human research participants
x		Clinical data

Methods

n/a	Involved in the study	
x		ChIP-seq
x		Flow cytometry
x		MRI-based neuroimaging

Eukaryotic cell lines

Policy information about cell lines

Cell line source(s)	VeroE6 (Peter Bredenbeek, LUMC, the Netherlands; ATCC® CRL-1586™)<br>HuH7 (JCRB Cell Bank JCRB0403)<br>ExpiCHO cells (ThermoFisher Scientific, A29127)<br>Calu-3 cells (Lieve Naesens, KU Leuven Rega Institute, Belgium; ATCC® HTB-55™)
Authentication	No authentication of the cell lines was performed.
Mycoplasma contamination	All cell lines tested negative for mycoplasma contamination.
Commonly misidentified lines (See ICLAC register)	None of the commonly misidentified cell lines were used.

Animals and other organisms

Policy information about studies involving animals; ARRIVE guidelines recommended for reporting animal research

Laboratory animals	Wild-type Syrian hamsters (Mesocricetus auratus) were purchased from Janvier Laboratories. All other mouse (C57BL/6, Ifnar1-/-, Il28r-/-, BALB/c and SCID) and hamster (STAT2-/- and IL28R-a-/-) strains were bred in-house. Six- to eight-weeks-old

	female mice and female wild-type hamsters were used throughout the study. Knock-out hamsters were used upon availability; seven- to twelve-week old female STAT2-/- hamsters; five- to seven-week-old IL28R-a-/- hamsters.
Wild animals	No wild animals were used in the study.
Field-collected samples	No field-collected samples were used in the study.
Ethics oversight	Housing conditions and experimental procedures were approved by the ethical committee of KU Leuven (license P015-2020), following institutional guidelines approved by the Federation of European Laboratory Animal Science Associations (FELASA).

Note that full information on the approval of the study protocol must also be provided in the manuscript.
