# Supplementary material for: STAT2 signaling restricts viral dissemination but drives severe pneumonia in SARS-CoV-2 infected hamsters
Source: Nat Commun. 2020 Nov 17;11:5838. doi: 10.1038/s41467-020-19684-y (PMC7672082; doi:10.1038/s41467-020-19684-y)
Supplement: Supplementary file 2 — Peer Review File [file 41467_2020_19684_MOESM2_ESM.pdf]

Editorial Note: This manuscript has been previously reviewed at another journal that is not operating a transparent peer review scheme. The manuscript was considered suitable for publication without further review at Nature Communications.
